# Irreversibility analysis for flow of nanofluids with aggregation in converging and diverging channel

**DOI:** 10.1038/s41598-022-14529-8

**Published:** 2022-06-17

**Authors:** Muhammad Qadeer, Umar Khan, Sarfraz Ahmad, Basharat Ullah, Mohamed Mousa, Ilyas Khan

**Affiliations:** 1 Department of Mathematics, Abbottabad University of Science and Technology, Abbottabad, Pakistan; 2 Department of Mathematics and Statistics, Hazara University, Mansehra, Pakistan; 3 Electrical Engineering, Faculty of Engineering and Technology, Future University in Egypt, New Cairo, 11835 Egypt; 4 Department of Mathematics, College of Science Al-Zulfi, Majmaah University, Al-Majmaah, P.O. Box 66, Majmaah, 11952 Saudi Arabia

**Keywords:** Applied mathematics, Computational science

## Abstract

In the current research article, the two-dimensional, incompressible, steady fluid flow is considered. The heat transfer rate of water-based aggregated fluid between converging/diverging channels of shrinking/stretching walls due to the effects of thermal radiation has been examined. The strong static magnetic field is applied perpendicular to the radial direction. The modeled governing equations are transformed into non-linear dimensionless ordinary differential equations by considering appropriate similarity transformations. Since the obtained ODEs are strongly non-linear and the exact solution of these equations is not possible, thus we applied the numerical method RK4 combined with the shooting technique to handle the equations. The impacts of several influential parameters on velocity, temperature, and entropy generation profiles are examined graphically.

## Introduction

A channel whose cross section decreases (increases) along the path of fluid flow until the minimum (maximum) area is obtained is known as convergent (divergent) channel. The incompressible, viscid 2D fluid flow between convergent/divergent channels whose walls are separated through fixed angle and determined by sink or source at the apex is defined as the Jaffery-Hamel flow. It has large number of applications in mechanical, civil, chemical, and aerospace engineering as well as in physical and biological fields. Blood flow through capillaries and arteries, flow of rivers and canals are also the examples of convergent/divergent channel flow. The innovative work about convergent/divergent flow was initiated by Jeffery and Shaw^1,2^.

Alam^3^ examined the impacts of incompressible MHD flow of copper nanoparticles on entropy generation by using joule heating effects and viscous dissipation passing through convergent/divergent channels. They investigated that by augmenting the values of volume fraction by nanoparticles, the values of Reynold’s number upsurges. They also observed the opposite behavior of flow in converging and diverging channels by giving different values to the influential parameters. The convergent/divergent channels of stretched walls were considered by Gerdroodbary et al.^4^ in which they discussed the effects of thermal radiations on Jaffery-Hamel flow. They used nonlinear boundary layer theory for the governing equations. They also discussed the skin friction and heat transferring phenomenon. They investigated the improvement in the temperature field by incrementing thermal radiation parameter. Asghar et al.^5^ considered MHD Jaffery-Hamel flow in converging/diverging channels due to the effects of boundary stresses. They used traction BCs to solve the governing equations. They developed some analytical scheme to obtain the solution of nonlinear 3rd ordered ODE’s. They observed that the boundary stresses support the boundary layers. They also examined the properties of slip and non-slip conditions of inertial flow. Khan et al.^6^ considered the stretched convergent/divergent channel and analyzed the effects of Soret and Dafour impacts on Jaffery-Hamel flow of 2nd grade fluid. They solved the problem numerically as well as analytically. For analytical solution they used HAM procedure and for numerical purpose they used RKF. The effects of different pertinent parameters on Nusselt number, skin fraction and Sherwood numbers are also discussed. The study of converging/diverging channels in case of MHD Jeffery-Hamel flow was considered by Asadullah et al.^7^. Several research articles about convergent/divergent channel are available in which various flow properties have been inspected under the effects of MHD and other external forces. Enough material about this topic is available in^8–11^ and the references therein.

Polymers are the complex structure of non-Newtonian fluids which exhibits rheological effects. Hydrogen, hydrocarbons, and different mixtures of carbon materials are used for the manufacturing of polymers. It has lot of applications in biological, chemical, and mechanical fields. In chemical engineering polymer films are used for the conservation/generation of energy. Different polymer structures are used for the transportation of small molecules through polymeric materials. In biological field polymers are used for the growth of multi-functional bio-interfaces, which are applicable to a range of biomedical applications. Formation of sensors, new optical and biomedical tools is also the applications of polymers. Polymers also play an important role to overcome the consumption of energy in case of turbulent flow.

The rheological effects of polymers with spherical solid structures known as micropolar fluids were debated by Nayak et al.^12^. They used nonlocal lubrication theory of fluids with microstructures and calculated the liquid draining rate of thin film between two solid smooth surfaces. They observed that for all film thicknesses down to zero, the proposed viscosity model of nonlocal lubrication theory has the best settlement with the experimental results. The thermal radiation effects of MHD micropolar nanofluids channel flow with porous walls was explored by Alizadeh et al.^13^. They inspected the properties of nanoparticles volume fraction parameter, micro-polar parameter, radiation parameter and magnetic parameter on temperature field, velocity field and Nusselt number. Khan^14^ analyzed the effects of shear stresses and the rate of heat transferring through the hybrid nano polymers (CuO-TiO_2_-polymers) by using novel models of hybrid nanoparticles. He also observed that the resistive effects due to permeability decreases in case of nano polymers with CuO as compared to the hybrid nano polymers (CuO-TiO_2_-polymers) and by increasing the values of vortex viscosity parameter, the angular velocity decreases. It is also noted that the change in angular velocity is more effective in case of hybrid nano polymers as compared to nano polymers. Nadeem et al.^15^ considered 3D micropolar fluid flow on Riga plate and observed the behavior of heat transferring and thermal conductivity through the fluid. They considered the flow through exponentially stretched surface. They divided their analysis in two parts i.e., PEST (Prescribed exponentially ordered surface temperature) and PEHF (prescribed exponentially ordered heat flux). They observed that by enhancing the radiation parameter both PEST and PEHF reduces. Iram et al.^16^ considered joule effects, viscous dissipation and 1st ordered chemical reaction in micropolar fluid flow and noticed the changes in thermal conductivity and concentration gradient. They perceived that for positive values of chemical reaction, the concentration field rises and vice versa. A number of studies^17–20^ on polymers are presented in which various aspects have been discussed.

Aggregates are the groups of large number of colloidal inertial particles. The addition of such aggregates shows significant role to the shear forces and thermal conductivity of the fluid flow. e.g., sedimentation of particles in oceans, separation of solid liquid particles, flocculation of cells etc. Flow characteristics of nanoparticles also enhances with aggregation. Brownian motion of the fluid particles plays a significant role in the development of aggregates.

Bao et al.^21^ calculated the viscosity of nanoparticles by stable molecular dynamics under the effects of nanoparticles aggregates on Green–Kubo equation. They observed that the nanoparticles viscosity enhances with the nanoparticle’s aggregation. Ritschel and Totsche^22^ considered 3D natural fluid flow in permeable system and observed the effects of micrometer size aggregates on flow regimes. They considered the networks of typical porous medium to model the soil aggregates. They observed that by developing the fundamental aggregation properties, the transporting properties of fluid flow from soil pore space upsurges. Thomas Kurobe et al.^23^ used the solutes like O_2_, CO_2_ mineral nutrients between environment and the aggregates and analyzed the heterogeneity of melted substances into the ambient water. They designated the fluid flow and the solute supply around sinking aggregates by resolving the governing and advection–diffusion equations numerically. Roberto Camassa et al.^24^ considered the matter aggregation in fluid system under the effects of gravitational forces. They observed that particles suspended in stratified fluid self assembles and makes large aggregates without adhesion. They also investigated that the particles possessing the same heights results in the attractive horizontal forces. They solved the system of equations numerically. A lot of material on aggregation is available in Ref.^25–30^.

As during any thermal process, a lot of energy losses due to which the production rate decreases. To improve the efficiency of any thermal system it is essential to search out the factors of energy losses. Entropy is the measure of uncertainty (energy losses) in a system. Entropy helps us to identify the factors that are responsible for energy losses. The second law of thermodynamics states that all the real processes in the universe are irreversible. Entropy generation is an important tool to measure the irreversibility of real systems.

Bejan^31^ examined several causes for entropy generation in thermal engineering where entropy production abolishes available work of a system. Bejan^32^ also investigated the entropy generation effects on fluid flow by considering the force convective heat transfer, temperature gradient and viscosity effects. Thacher^33^ considered the idea of entropy rate to examine the effectiveness of thermal electrical generators and heat pumping. Mukherjee et al.^34^ discussed the second law of thermodynamics for spinning flow in cylindrical pipe whose walls are at constant temperature. Carrington et al.^35^ discussed the causes of entropy generation by the heat and mass transferring for both inlet and outlet flows by using control volume method. The entropy generation effects on transparent plate in permeable medium along with solar radiations effects were analyzed by Dehsara et al.^36^. They discussed the results numerically. Shukla et al.^37^ considered Al_2_O_3_-Cu water-based hybrid nanofluid through stretching/contracting walls under the effects of thermal radiations; they noticed that the volume fraction of hybrid nanoparticles shows a significant role for reduction of entropy generation. For solving the modeled problem, they used PHAM technique. Many researchers discussed about entropy generation on MHD flow, whose detail can be seen in the articles^38–41^ and the references therein.

In the current study, we considered the two-dimensional aggregated water-based fluid between converging/diverging channel of stretching/shrinking walls. The steady and incompressible flow is considered under the effects of strong thermal radiative effects. The numerical scheme RK4 is implemented to obtain the solution of nonlinear ODEs. The Bruggeman model is used to obtain the thermal conductivity of aggregation. The effects of different parameters like radiation, volume fraction by nanoparticles, Reynolds number, sweep angle, stretching/shrinking parameter, Eckert number on velocity field, temperature field and entropy generation are discussed graphically.

## Formulation of governing equations

**Figure 1:**
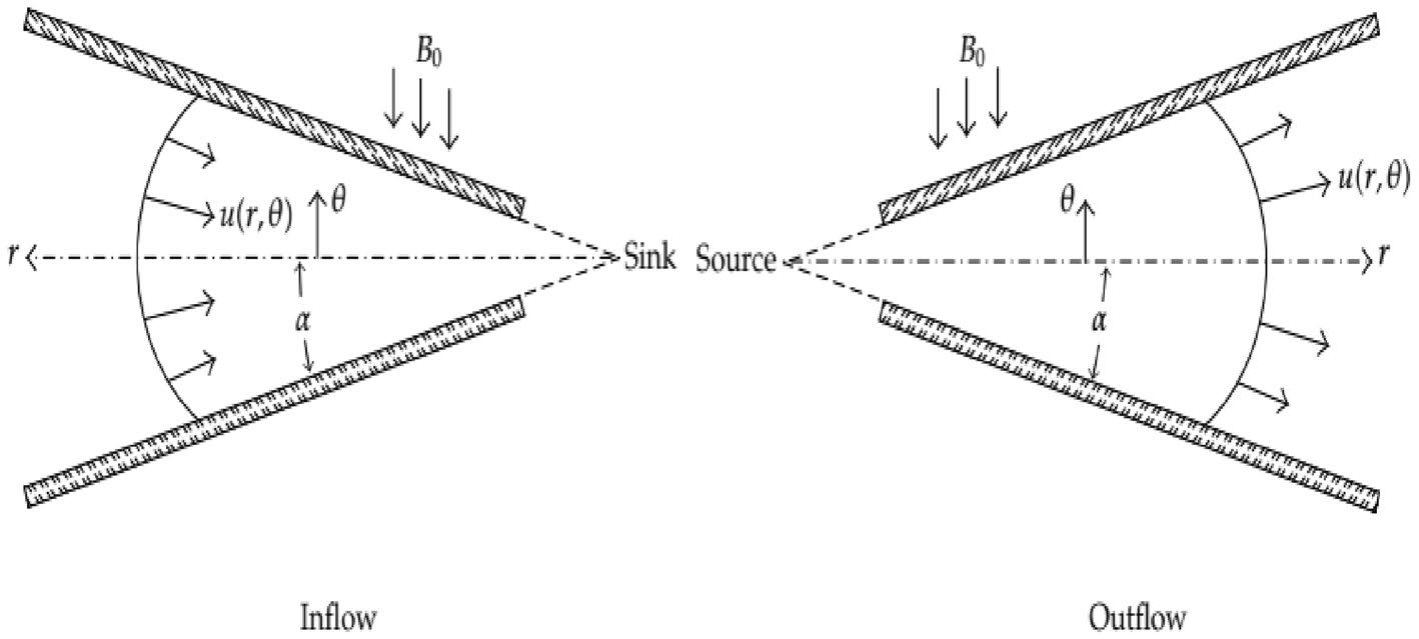
The schematic flow model through convergent (inflow) and divergent (outflow) channels.

Here we considered two-dimensional steady flow between converging/diverging channels. The non-parallel walls of the channel make a sweep angle of measurement $2\alpha$ at the intersection of walls. The cylindrical polar coordinate system is under consideration and the flow is measured only along the radial direction i.e., ${u}_{r}=[u\left(r,\Psi \right),0, 0]$. A static magnetic field ${B}_{0}$ is imposed in direction at right angle to ${u}_{r}$, Thus, the governing equations of fluid flow problem along with the appropriate boundary conditions are modeled as follows (Fig. 1)^4,42,43^,

1 $${{\varvec{\rho}}}_{{\varvec{a}}}\frac{1}{{\varvec{r}}}\left(\frac{\partial }{\partial {\varvec{r}}}\left({\varvec{r}}{\varvec{u}}\right)\right)=0,$$

2 $$u\frac{\partial u}{\partial r}+\frac{1}{{\rho }_{a}}\frac{\partial p}{\partial r}-{v}_{a}\left(\frac{{\partial }^{2}u}{{\partial r}^{2}}+\frac{1}{r}\frac{\partial u}{\partial r}+\frac{1}{{r}^{2}}\frac{{\partial }^{2}u}{{\partial \Psi }^{2}}-\frac{u}{{r}^{2}}\right)+\frac{{\sigma }_{a}}{{\rho }_{a}}{B}^{2}u=0,$$

3 $$r\frac{\partial p}{\partial \Psi }-2{\mu }_{a}\frac{\partial u}{\partial \Psi }=0,$$

4 $${u\frac{\partial T}{\partial r}-\frac{1}{{\left(\rho {C}_{p}\right)}_a}}k_a\left(\frac{1}{r}\frac{\partial T}{\partial r}+\frac{1}{{r}^{2}}\frac{{\partial }^{2}T}{{\partial \Psi }^{2}}+\frac{{\partial }^{2}T}{{\partial r}^{2}}\right)-\frac{1}{{\left(\rho {C}_{p}\right)}_{a}}{\mu }_{a}\left({\left(\frac{1}{r}\frac{\partial u}{\partial \Psi }\right)}^{2}+4{\left(\frac{\partial u}{\partial r}\right)}^{2}\right)-\frac{16\sigma {T}_{\infty }^{3}}{3{k}^{*}{\left(\rho {C}_{p}\right)}_{a}}\left(\frac{{\partial }^{2}T}{{\partial r}^{2}}+\frac{1}{r}\frac{\partial T}{\partial r}+\frac{1}{{r}^{2}}\frac{{\partial }^{2}T}{{\partial \Psi }^{2}}\right)=0$$

The appropriate BCs at shrinking/stretching walls are:

5 $$\frac{\partial u}{\partial \Psi }=0, \; u=U=\frac{{u}_{c}}{r}, \; \frac{\partial T}{\partial \Psi }=0 \; \; \text{at} \; \Psi =0.$$

6 $$u={u}_{w}=\frac{{s}_{t}}{r}, \;T=\frac{{T}_{w}}{{r}^{2}} \;\; \text{at} \;\Psi =\alpha .$$

To convert the above system into dimensionless form, the following similarity transformations are applied.

7 $$\eta =\frac{\Psi }{\alpha }, \; f\left(\eta \right)=\frac{F(\Psi )}{rU}, \; \theta \left(\eta \right)={r}^{2}\frac{T}{{T}_{w}}$$

In above Eqs. (1–7), ${u}_{w}$ is velocity at the wall of the channel, ${s}_{t}$ is stretching/shrinking parameter, $\alpha$ is sweep angle, ${u}_{c}$ represents the velocity of the particles along the centerline of channel, ${u}_{r}=(u\left(r,\Psi \right),0 , 0)$ is cylindrical form of velocity component along radial direction, $B$ is magnetic field parameter, ${T}_{w}$ represents temperature at the wall of channel, ${T}_{\infty }$ is ambient temperature, $\eta$ is angular coordinate in dimensionless form, $f\left(\eta \right)$ is dimensionless velocity, $\theta \left(\eta \right)$ is dimensionless temperature and $P$ is pressure. The symbols ${\rho }_{a}, {\sigma }_{a}, {v}_{a}, {\mu }_{a}, {k}_{a}, {\left(\rho {C}_{p}\right)}_{a}$ represent density, electrical conductivity, kinematic viscosity, dynamic viscosity, thermal conductivity, specific heat capacity respectively for the aggregated nanoparticles. Which is shown in Table 1.

**Table 1:** Physical properties and their mathematical relationship^39^.

${C}_{i}$	Properties	Without aggregation	With aggregation
${C}_{1}$	Electric Conductivity	$1+\frac{3\left(\frac{{\sigma }_{s}}{{\sigma }_{f}}-1\right)\phi }{\left(\frac{{\sigma }_{s}}{{\sigma }_{f}}+2\right)-\left(\frac{{\sigma }_{s}}{{\sigma }_{f}}-1\right)\phi }=\frac{{\sigma }_{nf}}{{\sigma }_{f}}$	$1+\frac{3\left(\frac{{\sigma }_{s}}{{\sigma }_{f}}-1\right){\phi }_{a}}{\left(\frac{{\sigma }_{s}}{{\sigma }_{f}}+2\right)-\left(\frac{{\sigma }_{s}}{{\sigma }_{f}}-1\right){\phi }_{a}}=\frac{{\sigma }_{a}}{{\sigma }_{f}}$
${C}_{2}$	Density	$\left(1-\phi \right)+\frac{{\rho }_{s}}{{\rho }_{f}}\phi =\frac{{\rho }_{nf}}{{\rho }_{f}}$	$\left(1-{\phi }_{a}\right)+{\phi }_{a}\frac{{\rho }_{s}}{{\rho }_{f}}=\frac{{\rho }_{a}}{{\rho }_{f}}$
${C}_{3}$	Kinematic viscosity	$\frac{1}{{(1-\phi )}^{2.5}}=$ $\frac{{\mu }_{nf}}{{\mu }_{f}}$	${\left(1-\frac{{\phi }_{a}}{{\phi }_{m}}\right)}^{{\phi }_{m}\left[\eta \right]}=\frac{{\mu }_{a}}{{\mu }_{f}}$
${C}_{4}$	Thermal conductivity	$\frac{\left(\left({k}_{s}+2{k}_{f}\right)-2\phi \left({k}_{f}-{k}_{s}\right)\right)}{\left({k}_{s}+2{k}_{f}\right)+\phi \left({k}_{f}-{k}_{s}\right)}=\frac{{k}_{nf}}{{k}_{f}}$	$\frac{\left(\left({k}_{a}+2{k}_{f}\right)-2{\phi }_{a}\left({k}_{f}-{k}_{a}\right)\right)}{\left({k}_{a}+2{k}_{f}\right)+{\phi }_{a}\left({k}_{f}-{k}_{a}\right)}=\frac{{k}_{a}}{{k}_{f}}$
${C}_{5}$	Specific heat capacity	$\left(1-\phi \right)+\phi \left(\frac{{\left(\rho {C}_{p}\right)}_{s}}{{\left(\rho {C}_{p}\right)}_{f}}\right)=\frac{{\left(\rho {C}_{p}\right)}_{nf}}{{\left(\rho {C}_{p}\right)}_{f}}$	$\left(1-{\phi }_{a}\right)+{\phi }_{a}\left(\frac{{\left(\rho {C}_{p}\right)}_{s}}{{\left(\rho {C}_{p}\right)}_{f}}\right)=\frac{{\left(\rho {C}_{p}\right)}_{a}}{{\left(\rho {C}_{p}\right)}_{f}}$

In the above Table 1$\phi$ is (nanoparticles volume fraction), ${\phi }_{a}={\phi \left(\frac{{r}_{a}}{{r}_{p}}\right)}^{3-D}$ is (volume fraction of aggregated nanoparticles). The symbols ${\rho }_{f}, {\sigma }_{f}, {v}_{f}, {k}_{f}, {{\mu }_{f}, \left(\rho {C}_{p}\right)}_{f}$ are density, electrical conductivity, dynamic viscosity, thermal conductivity, kinematic viscosity and specific heat capacity of nanoparticles respectively and ${\rho }_{s}, {\sigma }_{s}, {{\mu }_{s},v}_{s} , {k}_{s}, {\left(\rho {C}_{p}\right)}_{s}$ density, electrical conductivity, kinematic viscosity, dynamic viscosity, thermal conductivity and specific heat capacity of base fluid respectively. $D=1.8$ is (Fractal Index), $\frac{{r}_{a}}{{r}_{p}}=3.34$ is (ratio of radii of aggregates to nanoparticles), ${\phi }_{m}=0.605$ is (maximum volume fraction of nanoparticles), $\left[\eta \right]=2.5$ is (Einstein coefficients).

The Bruggeman model was used for transforming the Maxwell model to obtain the thermal conductivity of aggregation. The aggregated thermal conductivity model was displayed as^44^:

8 $$\frac{{k}_{a}}{{k}_{f}}=\frac{1}{4}\left[\left(3{\phi }_{in}-1\right)\frac{{k}_{s}}{{k}_{f}}+3\left(\left(1-{\phi }_{in}\right)-1\right)+{\left\{{\left(\left(3{\phi }_{in}-1\right)\frac{{k}_{s}}{{k}_{f}}+3\left(\left(1-{\phi }_{in}\right)-1\right)\right)}^{2}+8\frac{{k}_{s}}{{k}_{f}}\right\}}^\frac{1}{2}\right].$$

where $${\phi }_{in}={\left(\frac{{r}_{a}}{{r}_{p}}\right)}^{D-3}$$.

By the applications of transformations (7), Eqs. (1–5) becomes,

9 $${C}_{3}{f}^{{{\prime}}{{\prime}}{{\prime}}}\left(\eta \right)+4{\alpha }^{2}{C}_{3}f{^{\prime}}\left(\eta \right)+2{C}_{2}Re\alpha f\left(\eta \right){f}^{{\prime}}\left(\eta \right)=0,$$

10 $${\theta }^{{{\prime}}{{\prime}}}\left(\eta \right)+4{\alpha }^{2}\theta \left(\eta \right)+\left(\frac{PrEc\alpha }{{C}_{4}Re{C}_{3}}\right){C}_{4}\left(4{\alpha }^{2}{\left(f\left(\eta \right)\right)}^{2}+{\left({\theta }^{{\prime}}\left(\eta \right)\right)}^{2}\right)+2\frac{{C}_{5}}{{C}_{4}}{\alpha }^{2}Prf\left(\eta \right)\theta \left(\eta \right)+\frac{Nr}{{C}_{4}}\left({\theta }^{{{\prime}}{{\prime}}}\left(\eta \right)+4{\alpha }^{2}\theta \left(\eta \right)\right)=0.$$

The transformed dimensionless boundary conditions corresponding to Eqs. (5) and (6) are as follows:

11 $$f\left(0\right)=1, \; {f}^{{\prime}}\left(0\right)=0, \;f\left(1\right)=\chi ,$$

12 $${\theta }^{{\prime}}\left(0\right)=0, \;\theta \left(1\right)=1.$$

In Eqs. (9–12) the dimensionless parameters are: $\chi =\frac{{s}_{t}}{{u}_{c}}$ (non-dimensional stretching shrinking parameter), $f$ (non-dimensional velocity),

$\theta$ (Non-dimensional temperature), $Ec=\frac{{{u}_{c}}^{2}}{{c}_{f}{T}_{w}}$ (Eckert number) $Re=\frac{{u}_{c}\alpha }{{v}_{f}}$ (Reynolds number), $Pr=\frac{{u}_{c}{\left(\rho {C}_{p}\right)}_{f}}{{k}_{f}}$ (Prandtl number) and $Nr=\frac{16{\sigma }_{1}{\left(T\right)}_{\infty }^{3}}{3{\mathrm{k}}_{1}{\mathrm{k}}_{f}}$ (Radiation parameter).

## Entropy analysis

The rate of entropy generation for the recent problem by following Bejan^45^ is expressed as:

13 $${S}_{G}=\frac{{k}_{a}{r}^{4}}{{{T}_{w}}^{2}}\left[\left(1+\frac{16{\sigma }_{1}}{3{k}_{a}{k}_{1}}\right)\left\{{\left(\frac{\partial T}{\partial r}\right)}^{2}+{\left(\frac{1}{r}\frac{\partial T}{\partial \Psi }\right)}^{2}\right\}\right]+\frac{{\mu }_{a}{r}^{2}}{{T}_{w}}\left[4{\left(\frac{\partial u}{\partial r}\right)}^{2}+{\frac{1}{{r}^{2}}\left(\frac{\partial u}{\partial \Psi }\right)}^{2}\right]+\frac{{\sigma }_{a}{B}^{2}}{{T}_{w}}{u}^{2},$$

Using the similarity transformations (7) and Eq. (13), the entropy generation number ${N}_{g}$ takes the form:

14 $${N}_{g}={C}_{4}\left(1+\frac{Nr}{{C}_{4}}\right)\left\{4{\left(\theta \right)}^{2}+\frac{1}{{\alpha }^{2}}{\left(\theta {^{\prime}}\right)}^{2}\right\}+\frac{\alpha {C}_{3}}{{C}_{4}Re}PrEc\left\{4{\left(f\right)}^{2}+\frac{{\left(f{^{\prime}}\right)}^{2}}{{\alpha }^{2}}\right\}+\left\{\left(\frac{\alpha PrEc}{{C}_{5}Re}\right){\left(f\right)}^{2}\right\}$$

## Solution procedure

In the current exploration the modeled physical problem is based upon conservation laws which appear in the form of PDEs. These equations are changed into the set of highly coupled nonlinear ODEs. Since the transformed equations are highly nonlinear and exact solution of these coupled equations is not available. To overcome this issue, in our work, we applied a famous numerical scheme (RKF) combined with shooting iteration technique to compute the solutions of the transformed fluid flow problem. Solutions for temperature, velocity and the entropy generation fields are observed graphically, and the impacts of several influential parameters are examined.

## Results and discussions

This section is divided into three parts to study the variation in temperature, velocity, and entropy generation of fluid flow for both converging/diverging channels of shrinking/stretching walls under the effects of different parameters like sweep angle $(\alpha )$, volume fraction of aggregated nanoparticles ${(\phi }_{a})$, stretching/shrinking parameters $(\chi )$, Eckert number $(Ec)$ and the Reynolds number $(Re)$.

**Figure 2:**
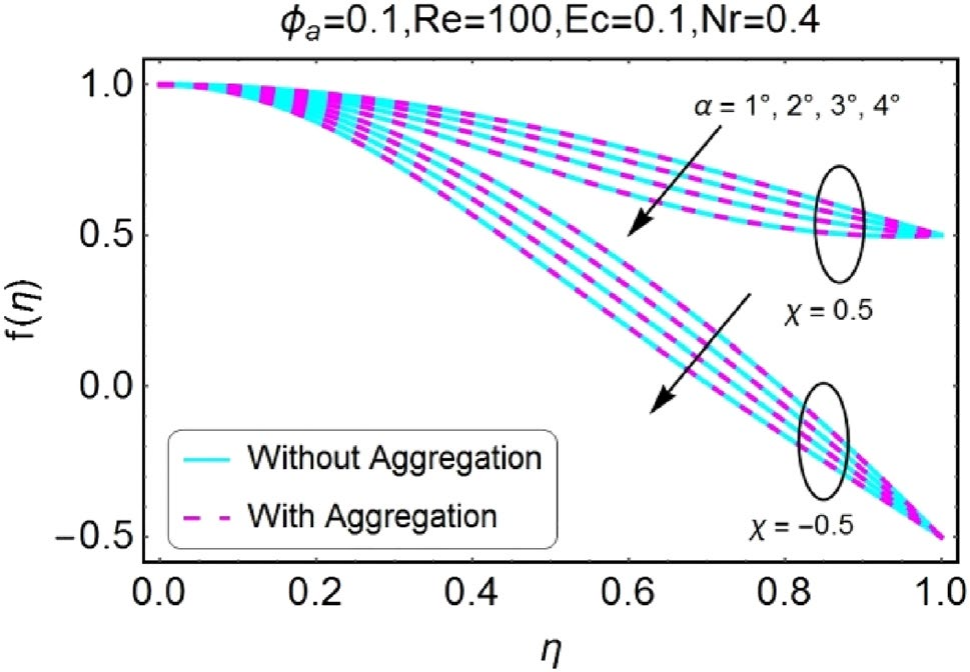
$\alpha$ varying $f$ for shrinking/stretching walls (divergent channel).

**Figure 3:**
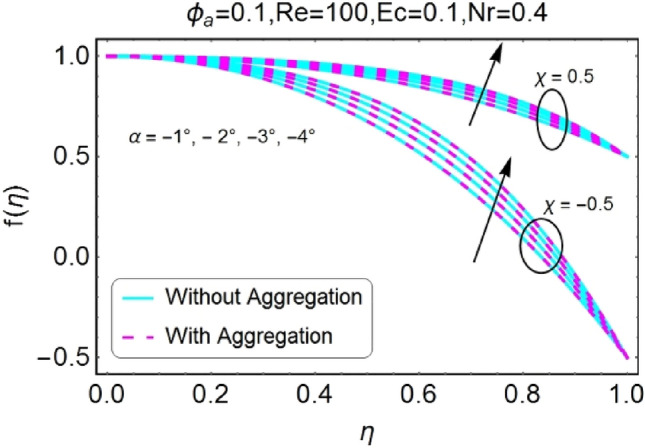
$\alpha$ varying $f$ for shrinking/stretching walls (convergent channel).

**Figure 4:**
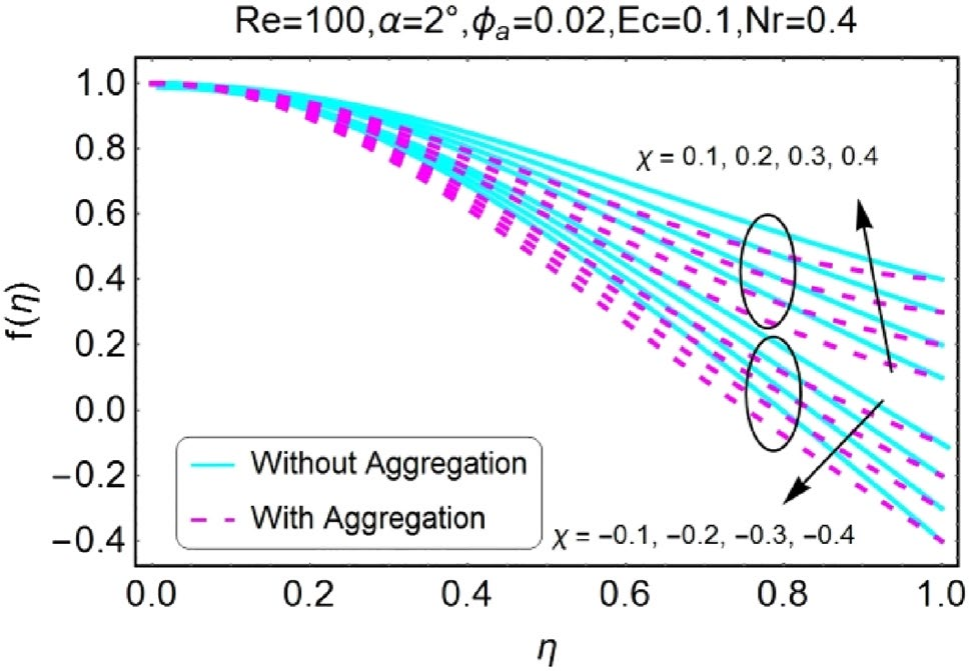
$\chi$ (shrinking/stretching parameter) varying $f$ (divergent channel).

**Figure 5:**
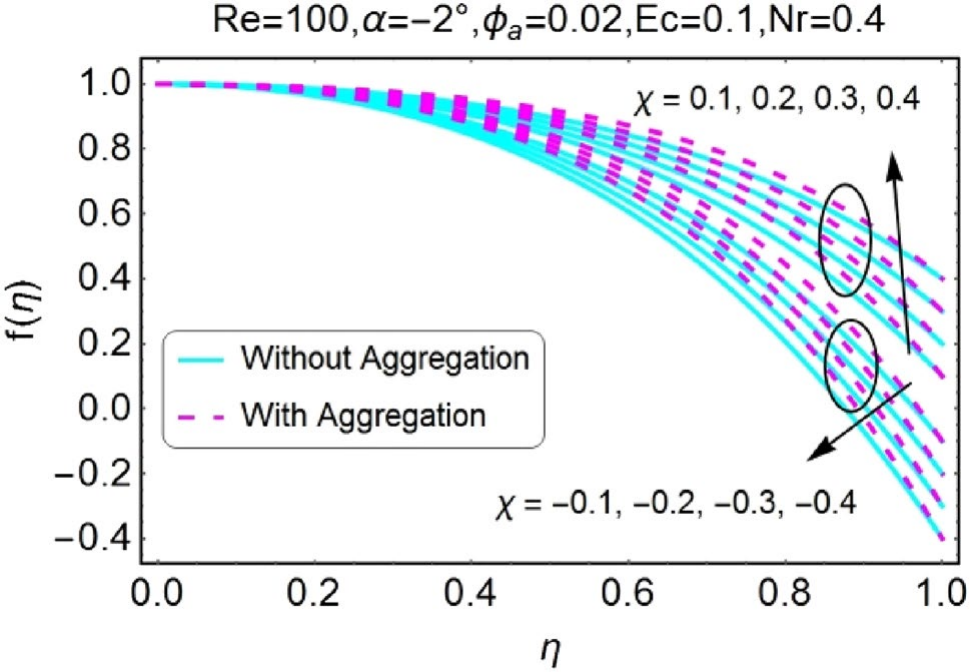
$\chi$ (shrinking/stretching parameter) varying $f$ (convergent channel).

**Figure 6:**
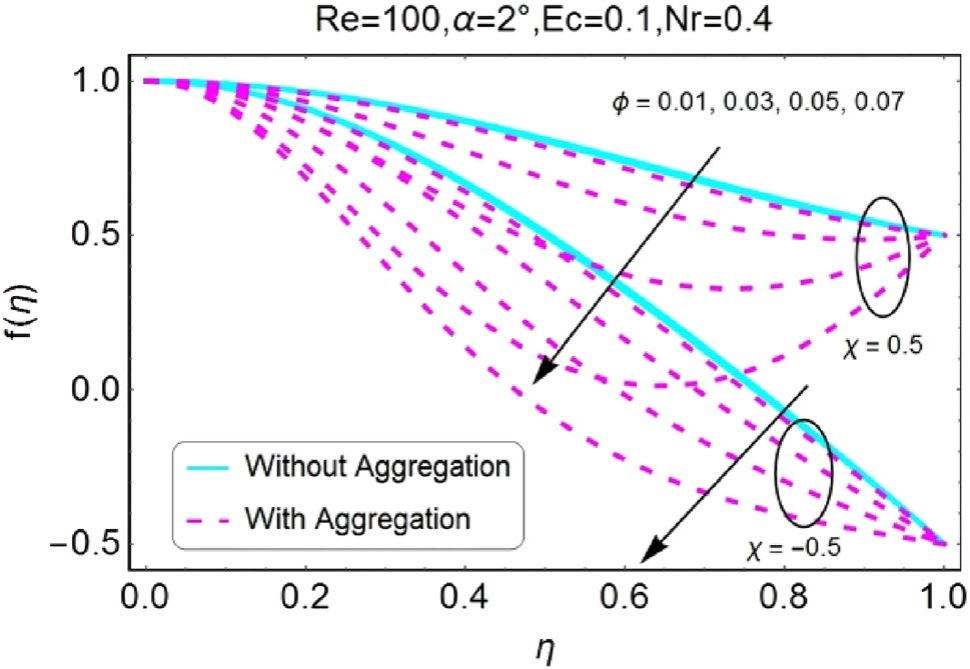
$\phi$ varying $f$ for shrinking/stretching walls (divergent channel).

**Figure 7:**
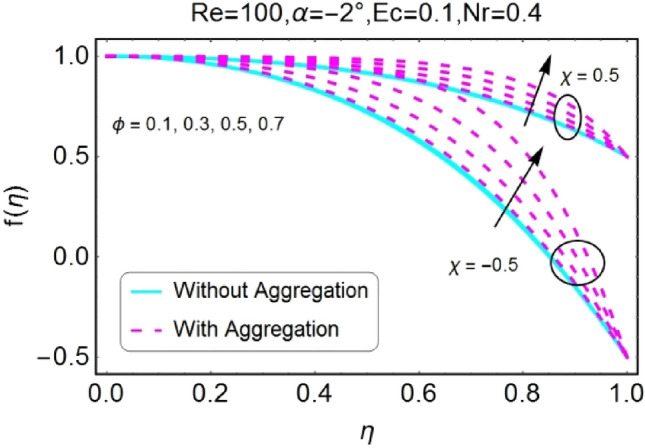
$\phi$ varying $f$ for shrinking/stretching walls (convergent channel).

**Figure 8:**
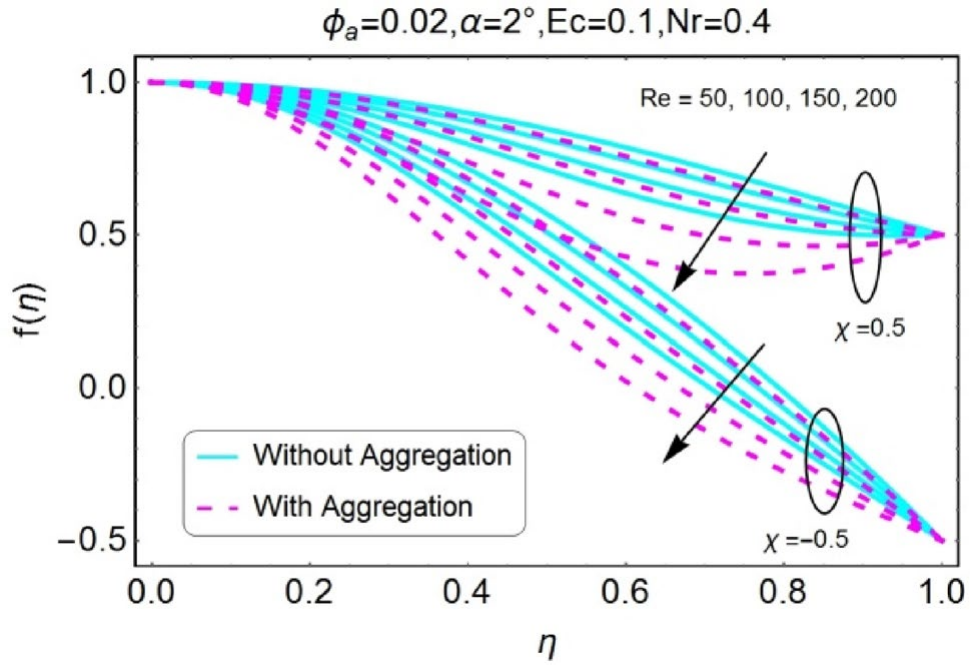
$Re$ varying $f$ for shrinking/stretching walls (divergent channel).

**Figure 9:**
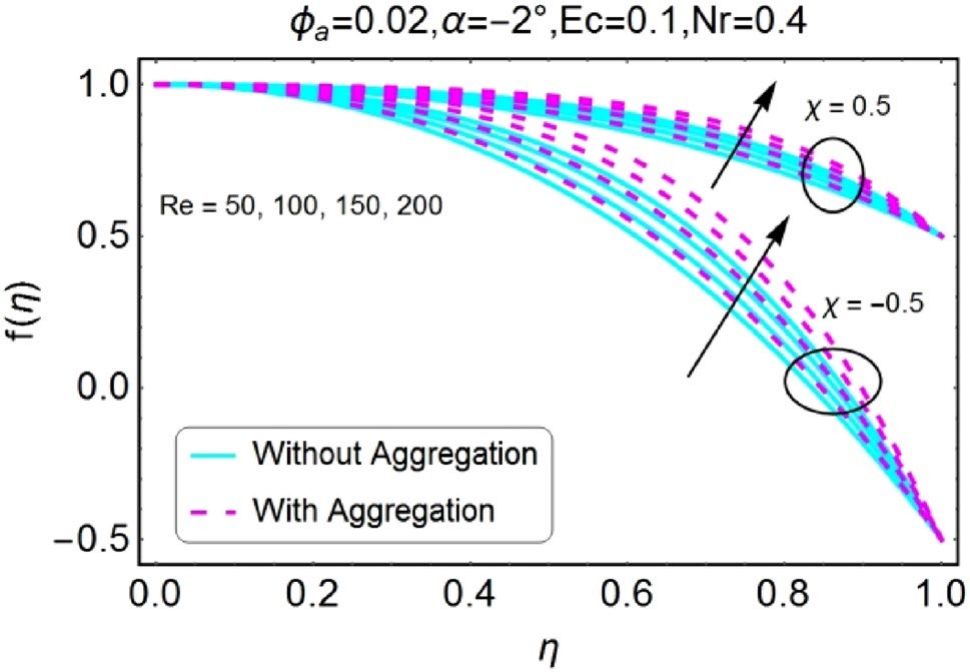
$Re$ varying $f$ for shrinking/stretching walls (convergent channel).

The impressions of pertinent parameters on velocity profile are plotted in set of Figs. 2, 3, 4, 5, 6, 7, 8, 9. Figure 2 shows that for the increasing values of opening angle $\alpha$ , the velocity profile decreases. Here the change is velocity is abrupt for the stretching wall near the central portion. An opposite impact of $\alpha$ on velocity is seen in Fig. 3 i.e., for the rise in angle $\alpha$, velocity profile upsurges for the converging channel and the alteration in velocity is on the lower side for stretching wall. The similar behavior is observed for both aggregation and non-aggregation models. The Figs. 4 and 5 are plotted for the variation in velocity due to the effects of stretching/shrinking parameter $\chi$. It is noted that for the stretching walls the velocity profiles rise for both converging and diverging channel. This rise in velocity closer to walls is more effective as that of central portion. While for shrinking walls, the quite opposite behavior for both converging and diverging channels is observed. The variation in velocity is more prominent near walls as related to center portion as shown in Figs. 4 and 5. Moreover the velocity of non-aggregated nanoparticles is slightly higher for the case of diverging channel and a reverse impact is noticed for converging channel. The change in velocity field for increasing values of volume fraction by nanoparticles is plotted in Figs. 6 and 7. The decline in velocity is perceived for the rising values of volume fraction by nanoparticles as shown in Fig. 6. The dominant rise in velocity of stretching wall is observed as likened to shrinking walls. The change in velocity for non-aggregation model is negligible as compared to aggregation model. All the effects of $\phi$ (nanoparticles volume fraction) on velocity in the situation of converging channel are quite opposite as related to divergent channel, except the seen that the change is prominent near centre as compared to walls as shown in Fig. 7. Figures 8 and 9 illustrate the impact of Reynolds number (Re) on velocity. It is seen that that the velocity decreases with the growing values of Reynolds number (Re) for stretching/shrinking diverging channel. The rapid change is observed closer to middle part of channel. This alteration in velocity is dominant for aggregated nanoparticles as compared to non-aggregated nanoparticles. The effects of Re on velocity for convergent shrinking/stretching is quite opposite to that of divergent shrinking/stretching channel. This change in the central compartment is more effective as compared to walls.

The change in the temperature field due to the impacts of different parameters like opening angle $\alpha$, nanoparticles volume fraction ${\phi }_{a}$, Reynolds number $Re$, Eckert number $Ec$ of both divergent and convergent channels are plotted in Figs. 10, 11, 12, 13, 14, 15, 16, 17, 18, 19, 20, 21, 22, 23, 24, 25, 26, 27, 28, 29, 30, 31, 32, 33). The belongings of stretching and shrinking walls are also discussed for both converging and diverging channels. The fixed value of $Pr$ is taken as 6.2. The impact of opening angle $\alpha$ on temperature profile is plotted in the set of Figs. 10, 11, 12, 13. Almost similar performance of temperature is observed for mounting values of $\alpha$ for both converging and diverging channels. For stretched walls the temperature profile is slightly above as related to shrinking walls. The similar properties of $\alpha$ for both aggregation and non-aggregation models are observed.

**Figure 10:**
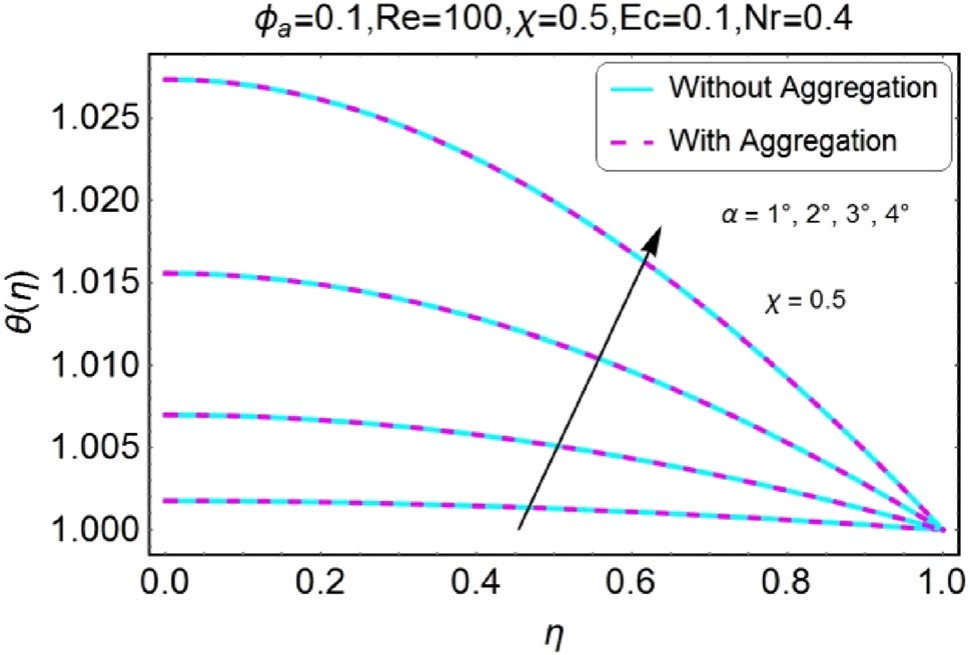
$\alpha$ varying $\theta$ for stretching walls (divergent channel).

**Figure 11:**
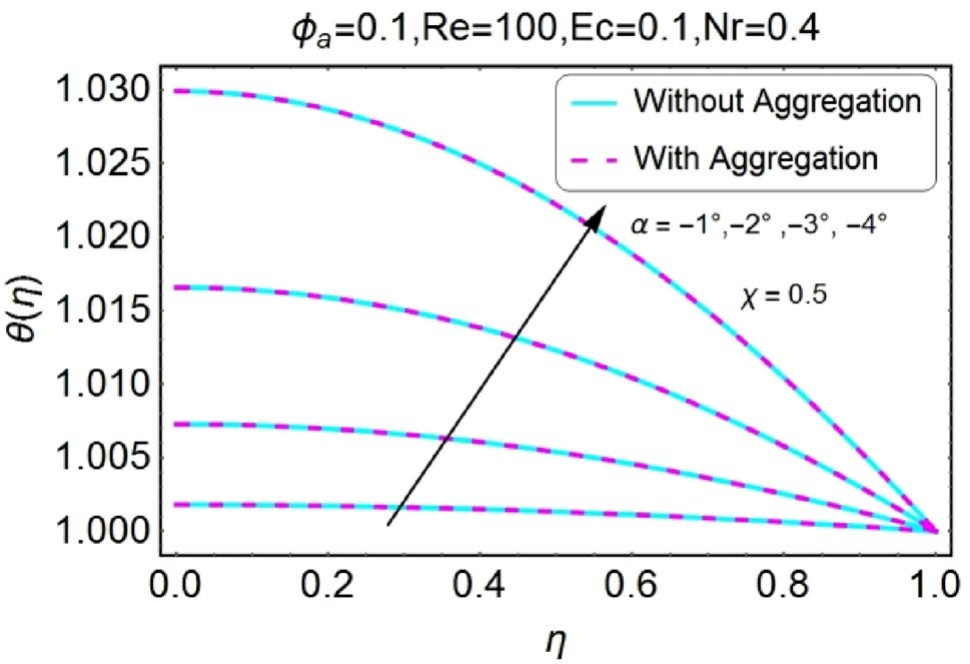
$\alpha$ varying $\theta$ for stretching walls (convergent channel).

**Figure 12:**
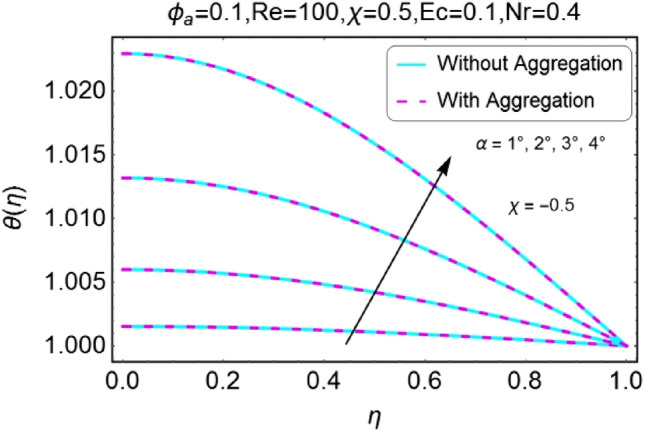
$\alpha$ varying $\theta$ for shrinking walls (divergent channel).

**Figure 13:**
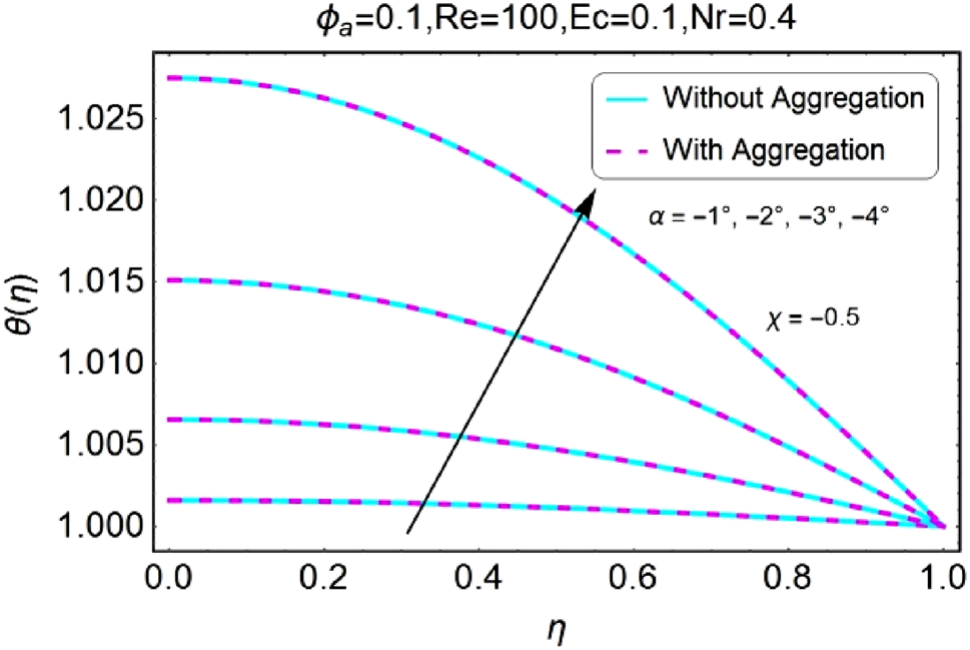
$\alpha$ varying $\theta$ for shrinking walls (convergent channel).

**Figure 14:**
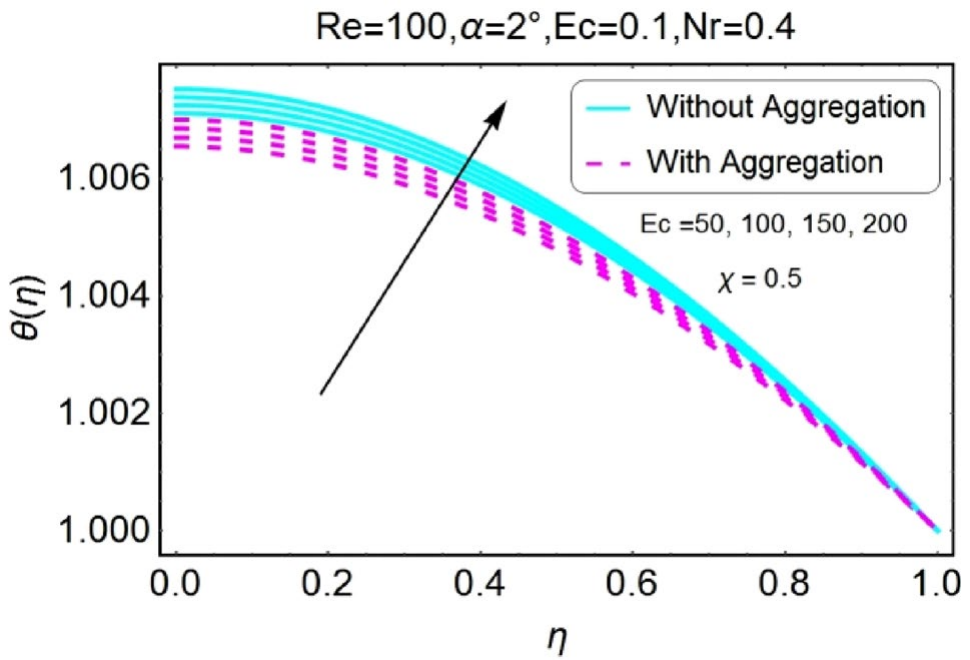
$Ec$ varying $\theta$ for stretching walls (divergent channel).

**Figure 15:**
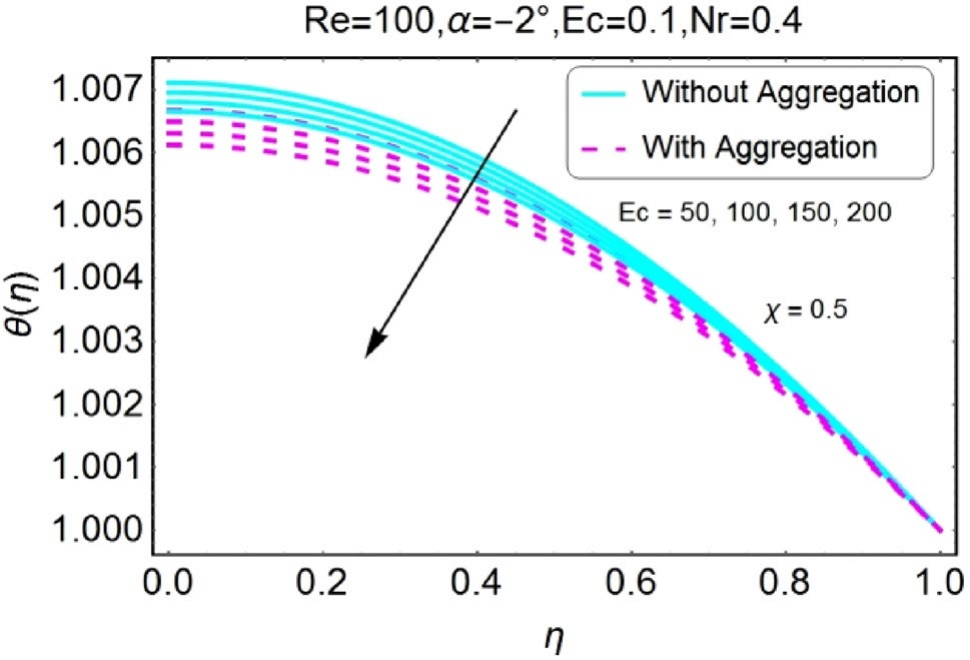
$Ec$ varying $\theta$ for stretching walls (convergent channel).

**Figure 16:**
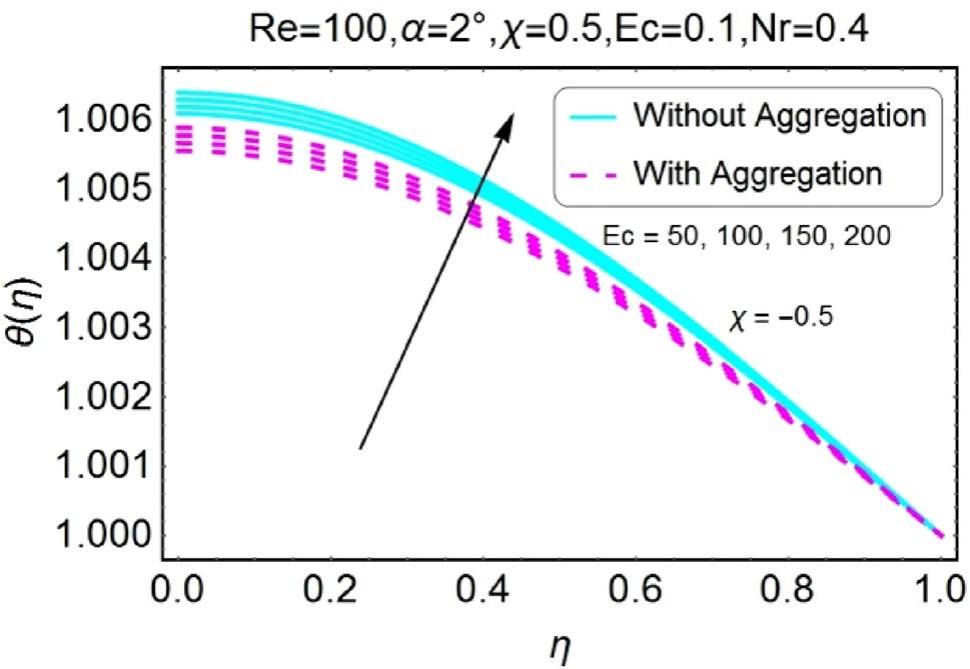
$Ec$ varying $\theta$ for shrinking walls (divergent channel).

**Figure 17:**
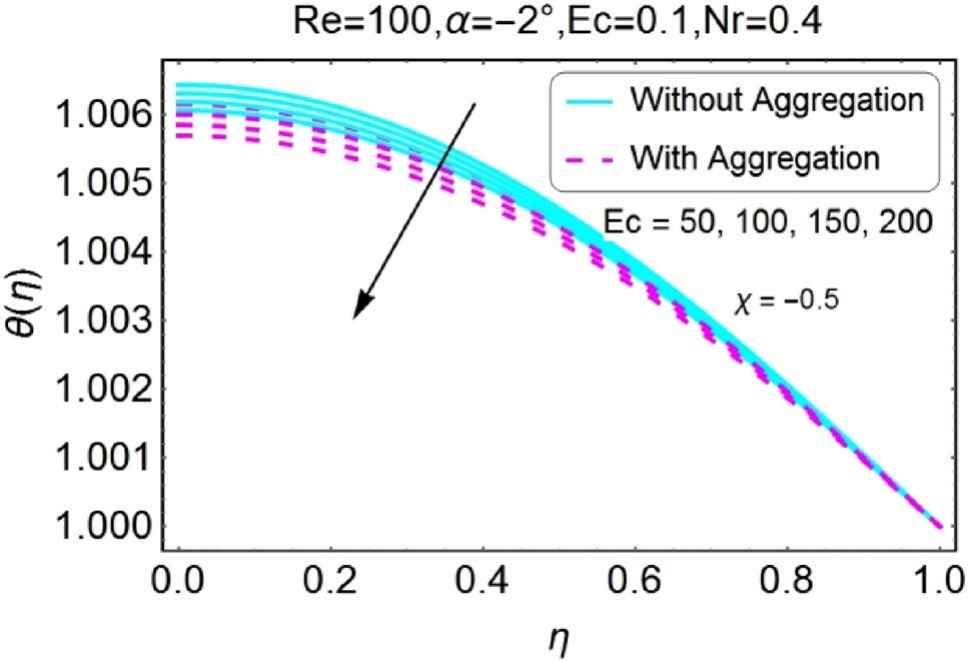
$Ec$varying $\theta$ for shrinking walls (convergent channel).

**Figure 18:**
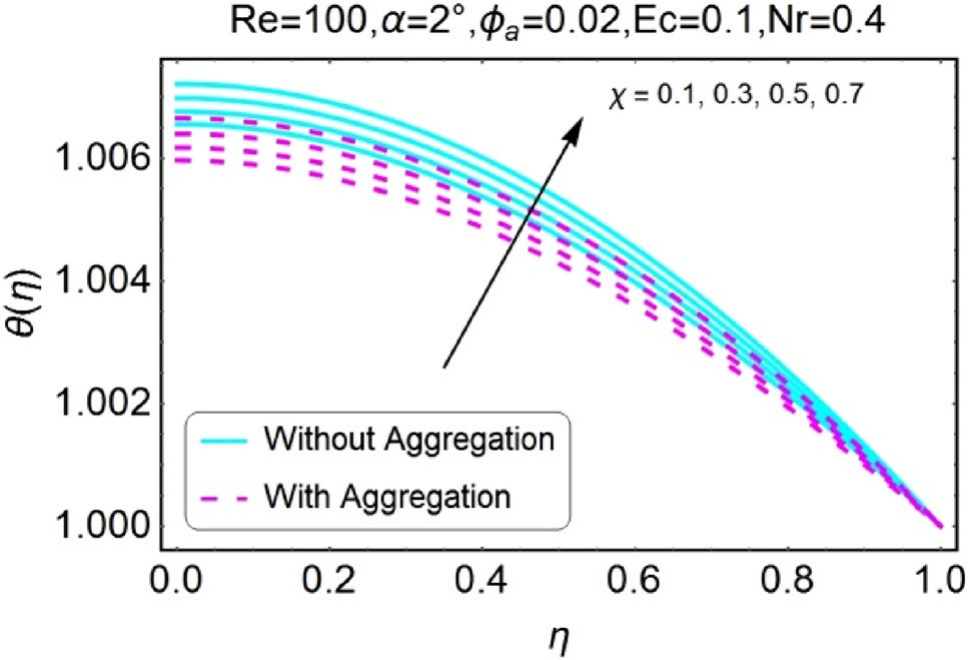
$\chi$ varying $\theta$ for stretching walls (divergent channel).

**Figure 19:**
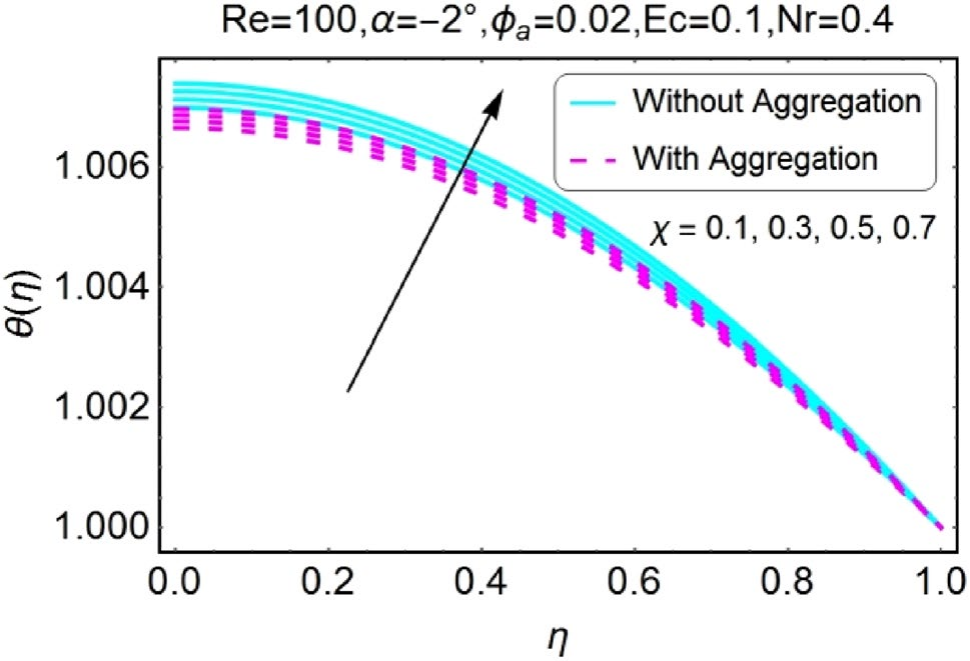
$\chi$ varying $\theta$ for stretching walls (convergent channel).

**Figure 20:**
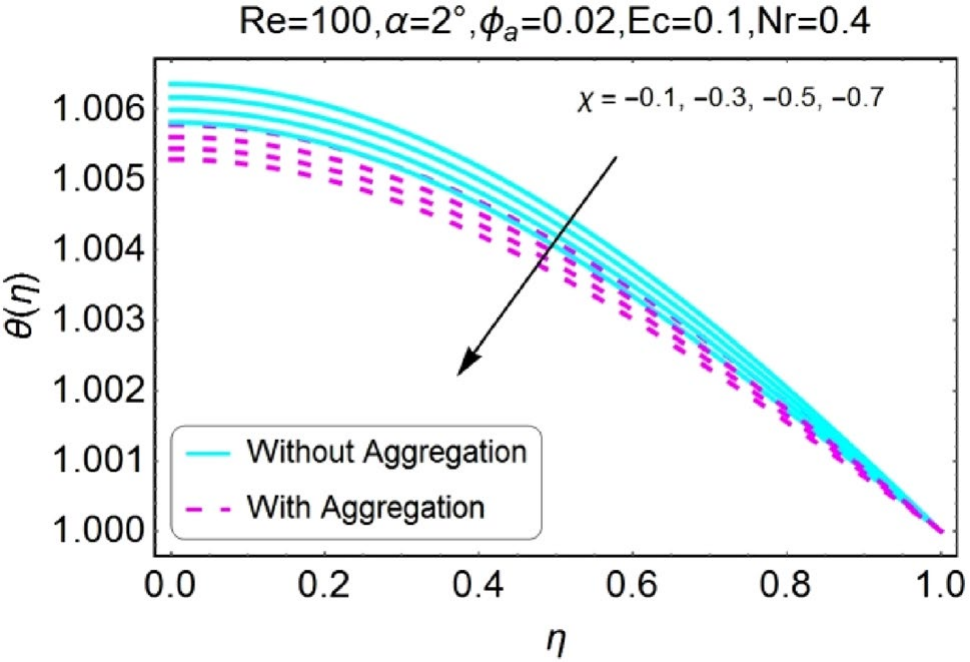
$\chi$ varying $\theta$ for shrinking walls (divergent channel).

**Figure 21:**
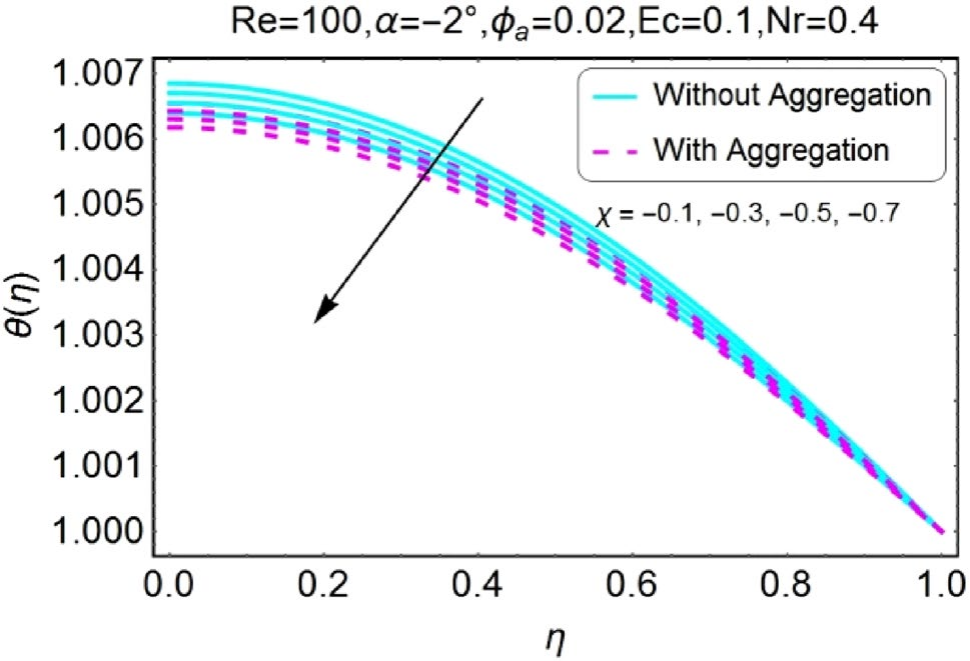
$\chi$ varying $\theta$ for shrinking walls (convergent channel).

**Figure 22:**
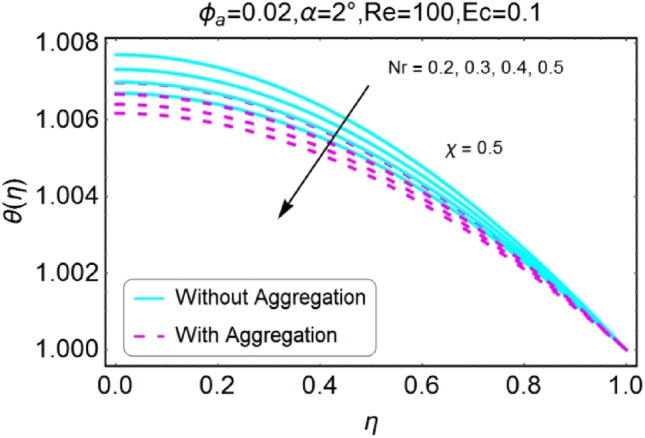
$Nr$ varying $\theta$ for stretching walls (divergent channel).

**Figure 23:**
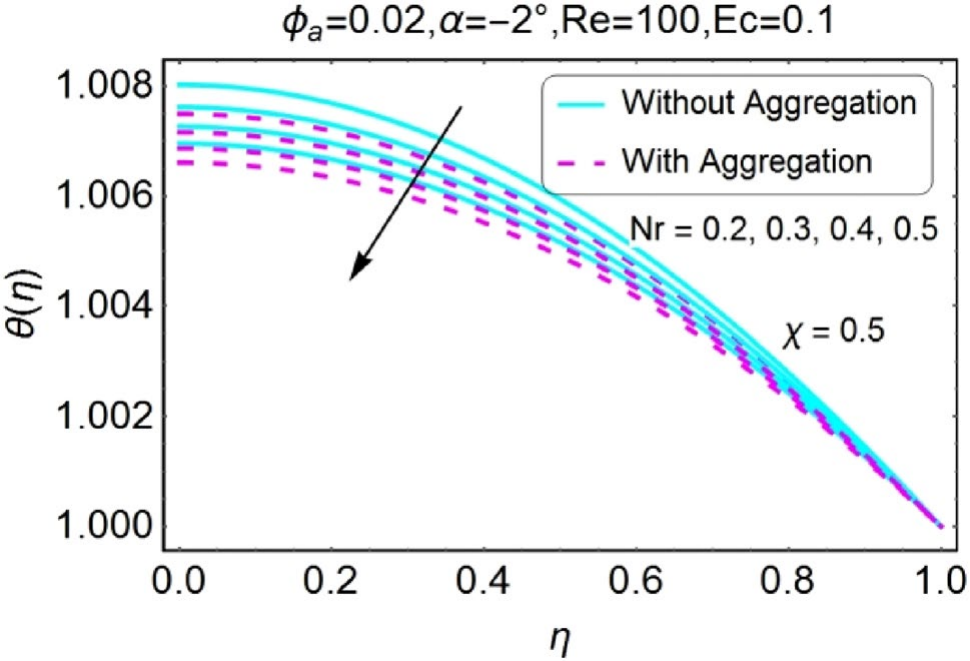
$Nr$ varying $\theta$ for stretching walls (convergent channel).

**Figure 24:**
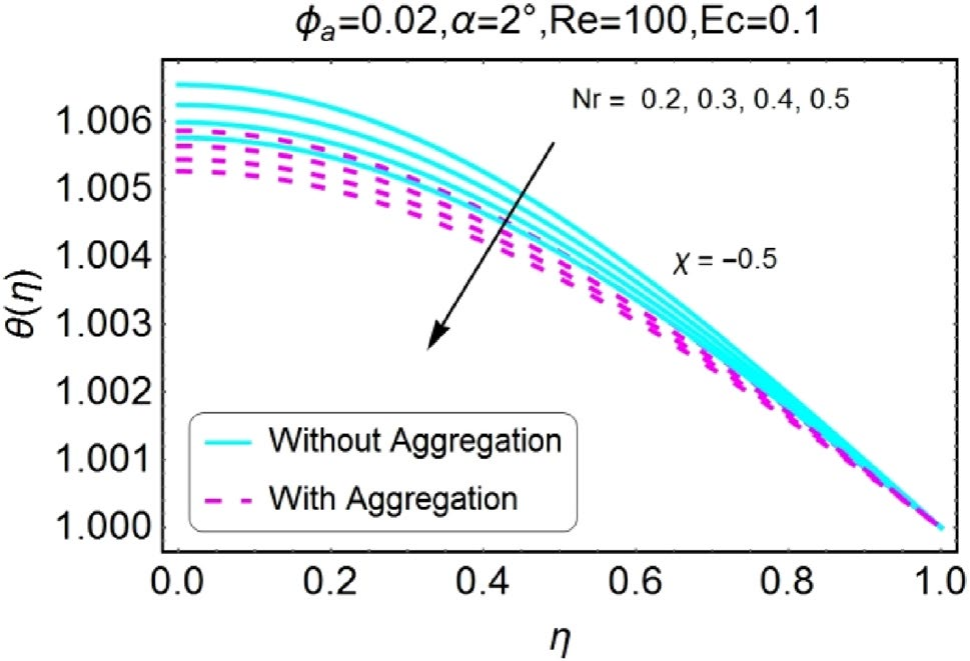
$Nr$ varying $\theta$ for shrinking walls (divergent channel).

**Figure 25:**
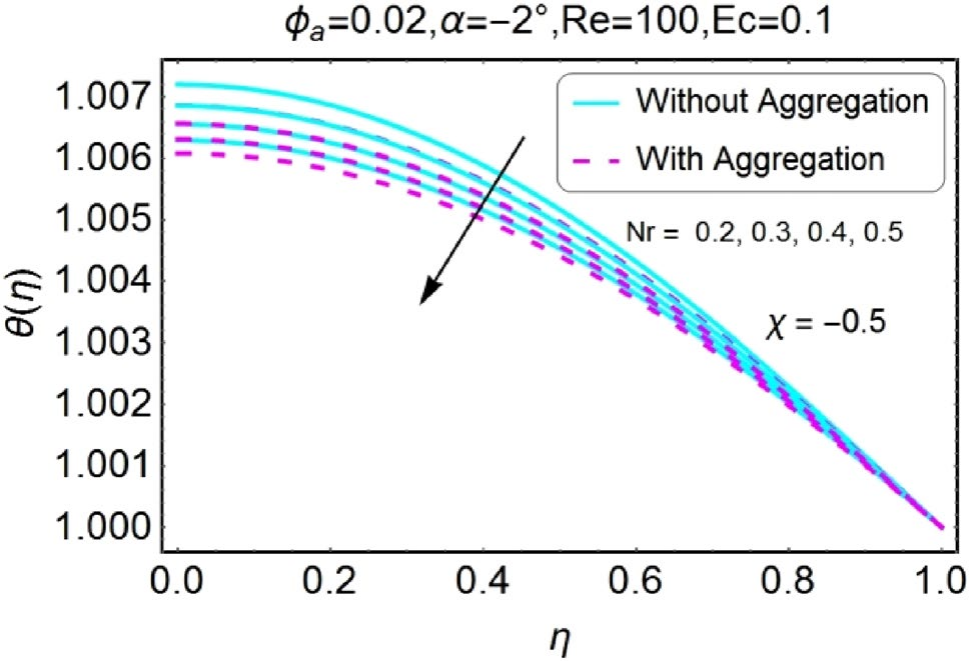
$Nr$ varying $\theta$ for shrinking walls (convergent channel).

**Figure 26:**
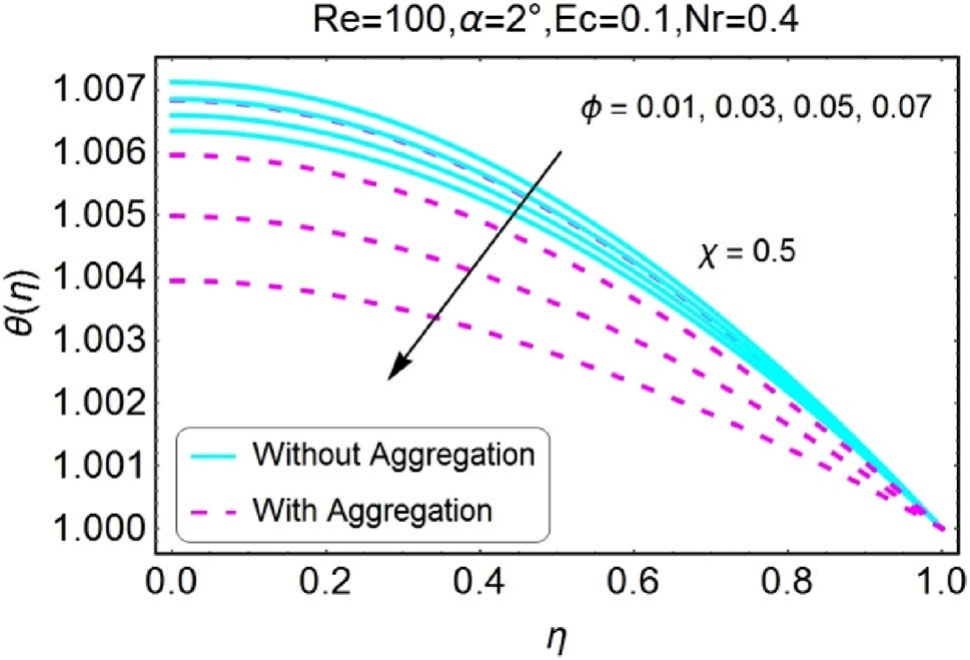
$\phi$ varying $\theta$ for stretching walls (divergent channel).

**Figure 27:**
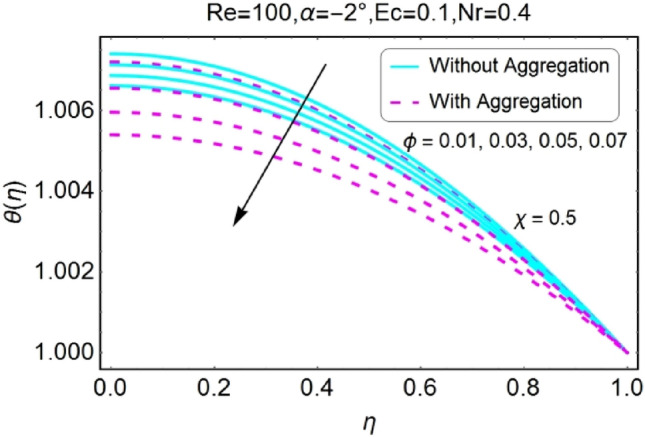
$\phi$ varying $\theta$ for stretching walls (convergent channel).

**Figure 28:**
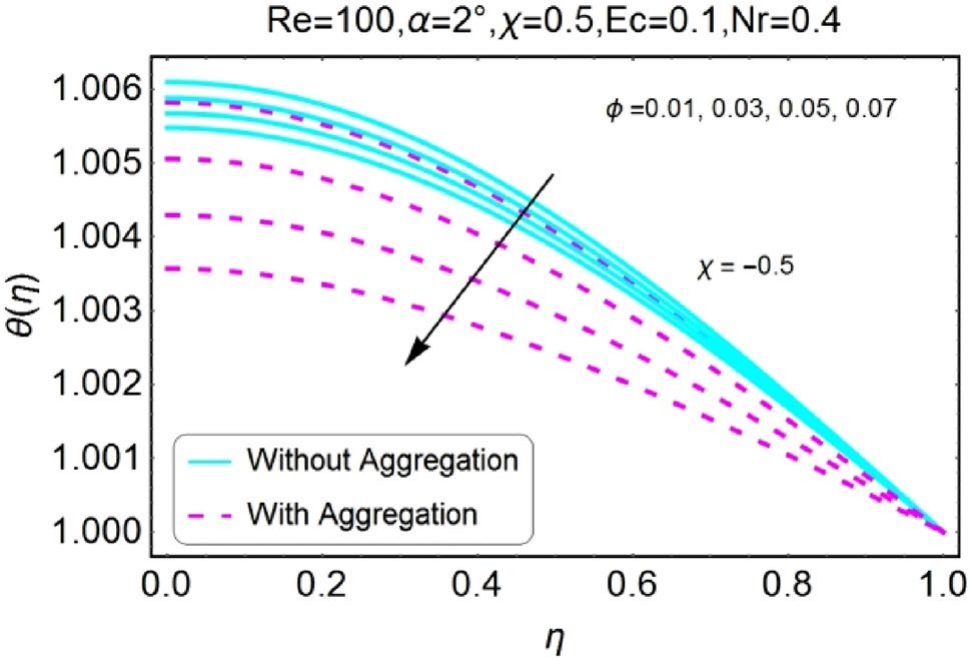
$\phi$ varying $\theta$ for shrinking walls (divergent channel).

**Figure 29:**
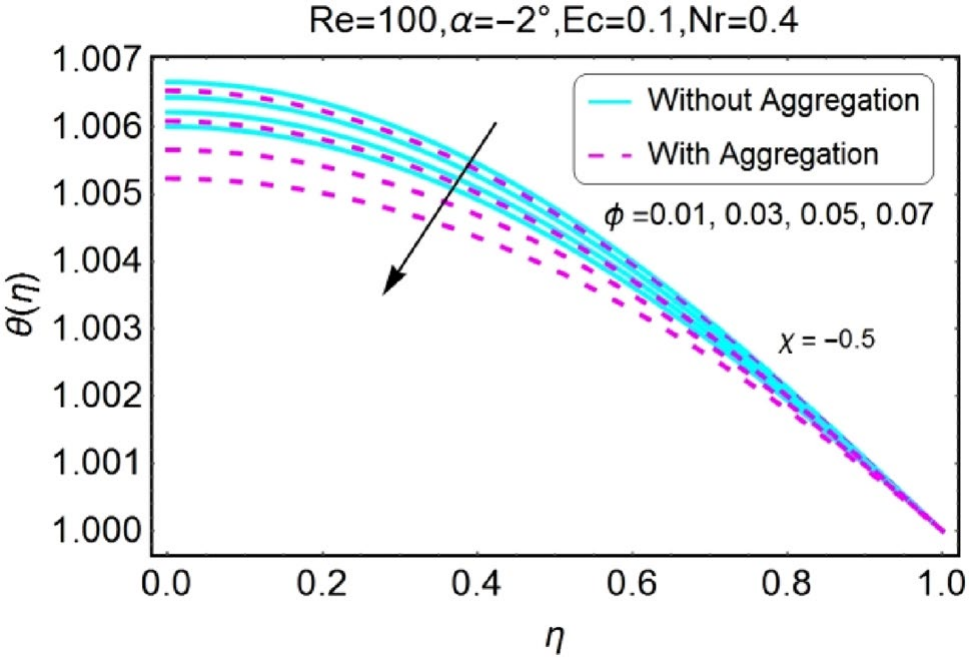
$\phi$ varying $\theta$ for shrinking walls (convergent channel).

**Figure 30:**
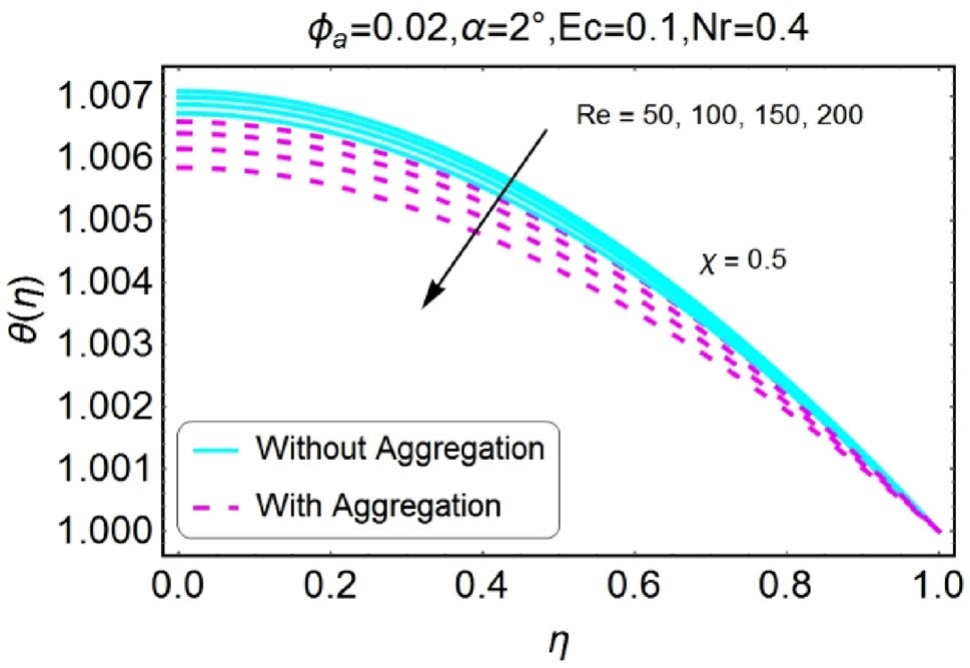
$Re$ varying $\theta$ for stretching walls (divergent channel).

**Figure 31:**
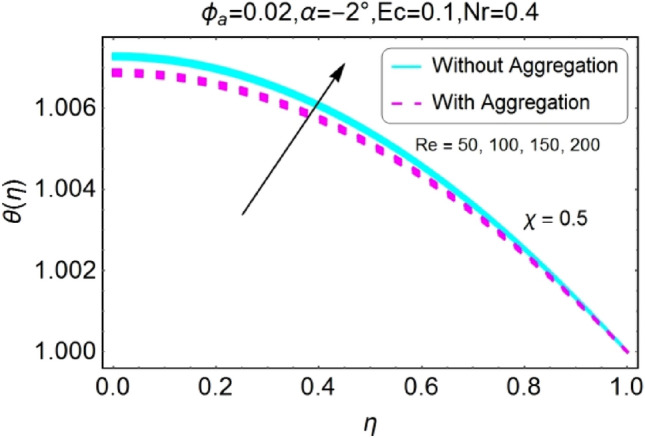
$Re$ varying $\theta$ for stretching walls (convergent channel).

**Figure 32:**
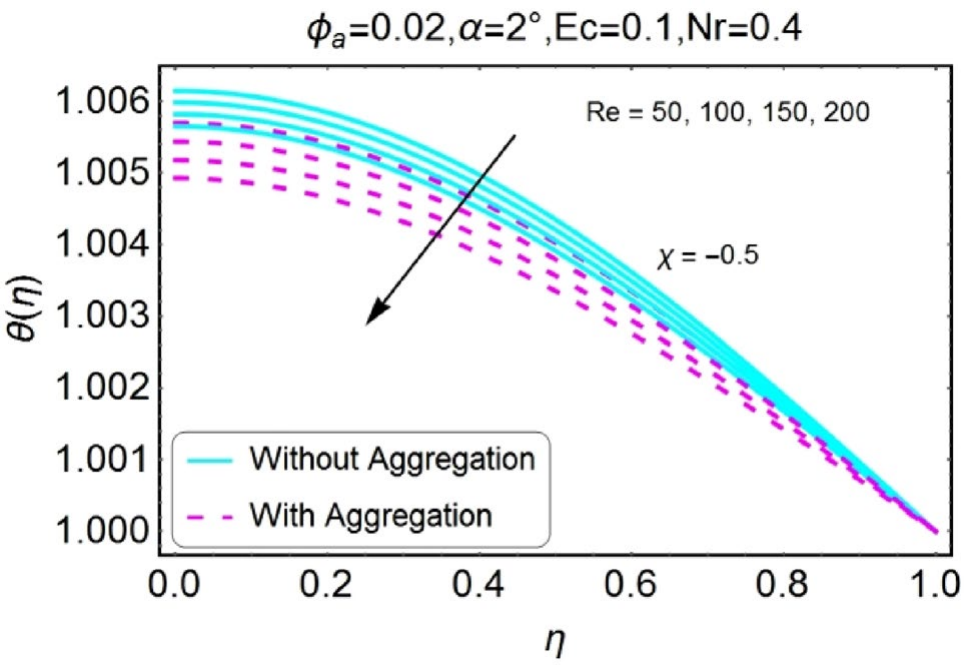
$Re$ varying $\theta$ for shrinking walls (divergent channel).

**Figure 33:**
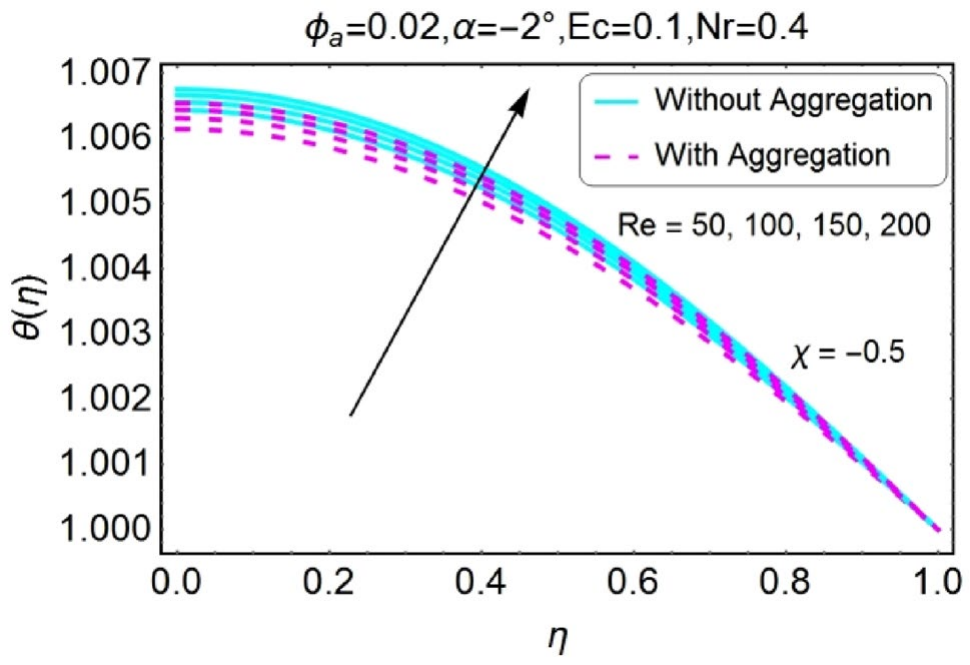
$Re$ varying $\theta$ for shrinking walls (convergent channel).

The impressions of Eckert number $Ec$ on temperature field is designed in the set of Figs. 14, 15, 16, 17. It is investigated that the temperature field upsurges with the mounting values of $Ec$ for both stretching and shrinking walls of diverging channel as shown in Figs. 14 and 16, whereas the temperature decreases for the converging channel of stretching/shrinking walls with growing values of $Ec$ as displayed in Figs. 15 and 17. The temperature profile for aggregated model is slightly lower as compared to non-aggregated model.

The variation of temperature profile due to stretching and shrinking parameter $\chi$ is shown in the set of Figs. 18, 19, 20, 21. The temperature profile rises for converging as well as diverging channels due to stretching parameter ($\chi >0$) as shown in Figs. 18 and 19. The opposite performance is found in case of shrinking parameter ($\chi <0$) i.e. temperature field declines as shown in Figs. 20 and 21. It is also noted that the rapid change is temperature is found for the divergent channel as compared to convergent channel.

The Figs. 22, 23, 24, 25 are portrayed to measure the change in temperature due to growing values of radiation parameter $Nr$. Almost similar behavior for all the cases i.e., stretching/divergent, shrinking/divergent, stretching/convergent, and shrinking/convergent is observed. For the rising values of radiation parameter ($Nr$), temperature profile declines. It is also noted that the change for diverging channel is rapid as likened to convergent channel. For all cases, the temperature profile for non-aggregated model is slightly above as compared to aggregated model.

The Impact of volume fraction by nanoparticles on temperature field is designed in the set of Figs. 26, 27, 28, 29. The temperature profile increases for growing values of ${\phi }_{a}$ for all the cases i.e., stretching/divergent, shrinking/divergent, stretching/convergent, and shrinking/convergent. Moreover, the rapid change in temperature profile for aggregated model is observed as compared to nan-aggregated model.

The opposite impact of Reynolds number on temperature field is observed for diverging and converging channels as displayed in Figs. 30, 31, 32, 33. For divergent channel the declining effect of temperature profile is perceived due to growing values of Reynolds number for stretching/shrinking walls. On the other hand, the temperature field for converging channel rises for stretching/shrinking walls. This change in temperature for aggregation model is rapid as compared to non-aggregation model.

**Figure 34:**
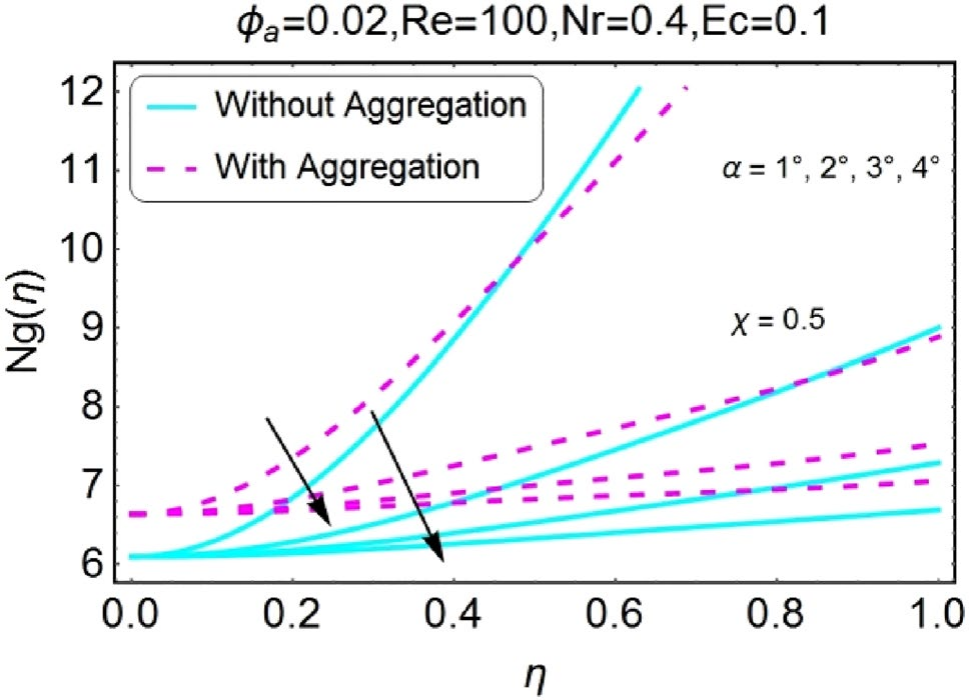
$\alpha$ varying entropy for stretching walls (divergent channel).

**Figure 35:**
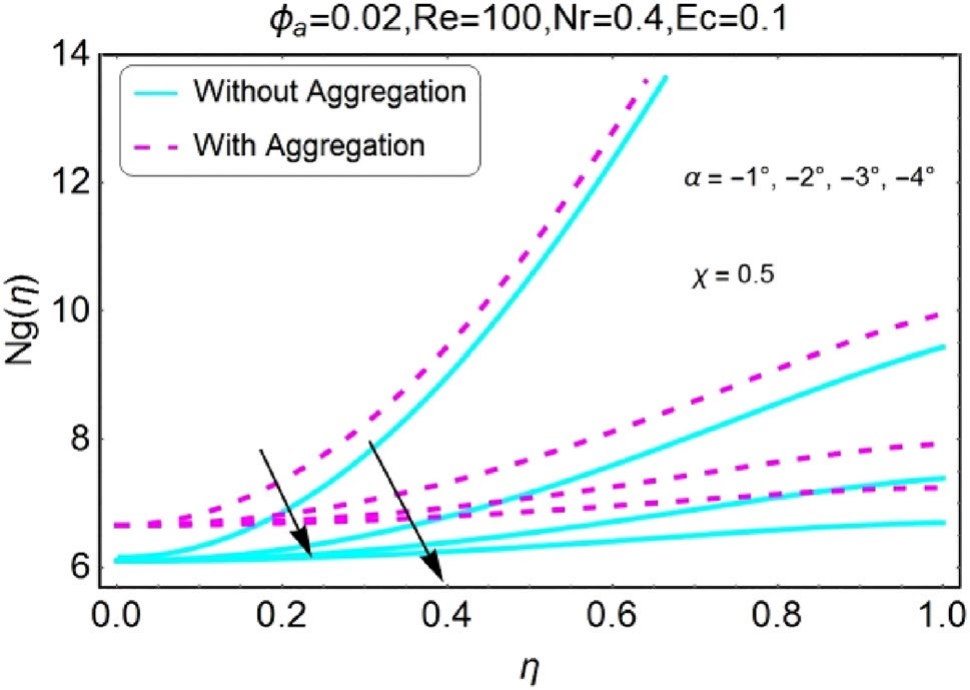
$\alpha$ varying entropy for stretching walls (convergent channel).

**Figure 36:**
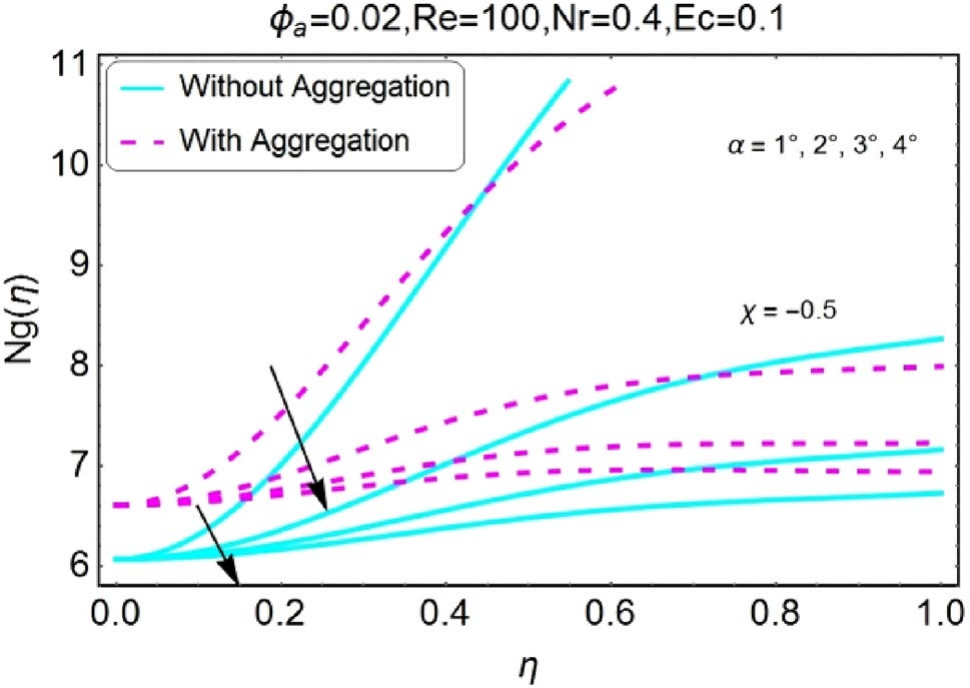
$\alpha$ varying entropy for shrinking walls (divergent channel).

**Figure 37:**
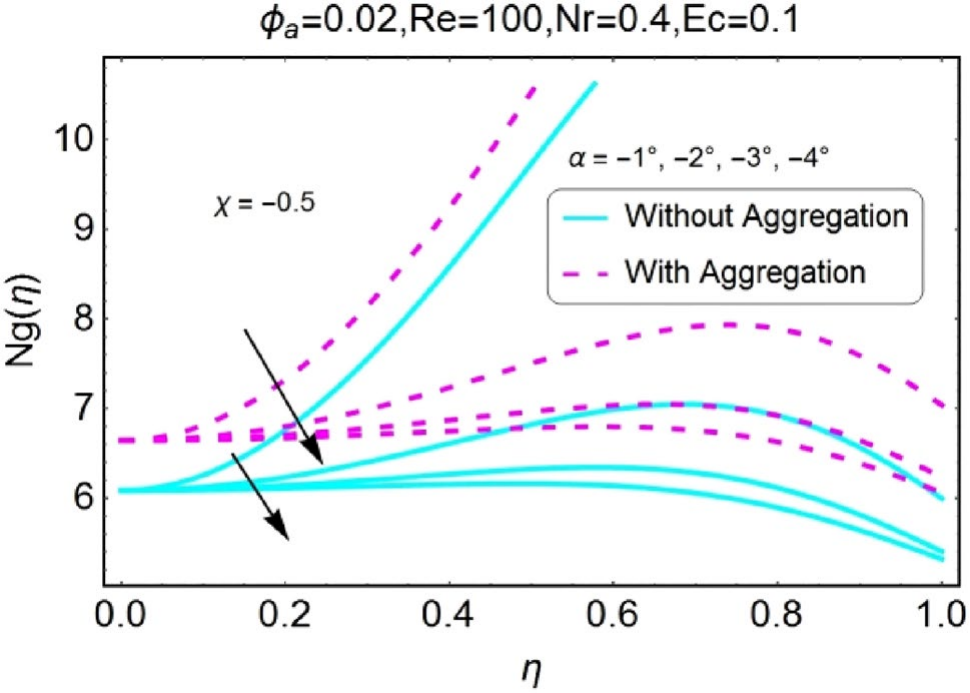
$\alpha$ varying entropy for shrinking walls (convergent channel).

**Figure 38:**
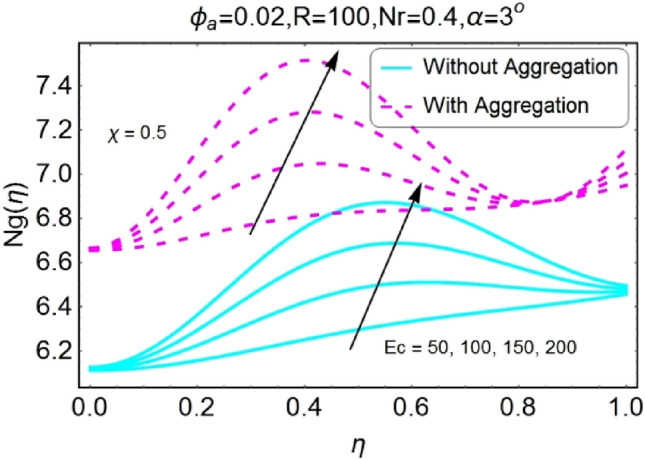
$Ec$ varying $Ng$ for stretching walls (divergent channel).

**Figure 39:**
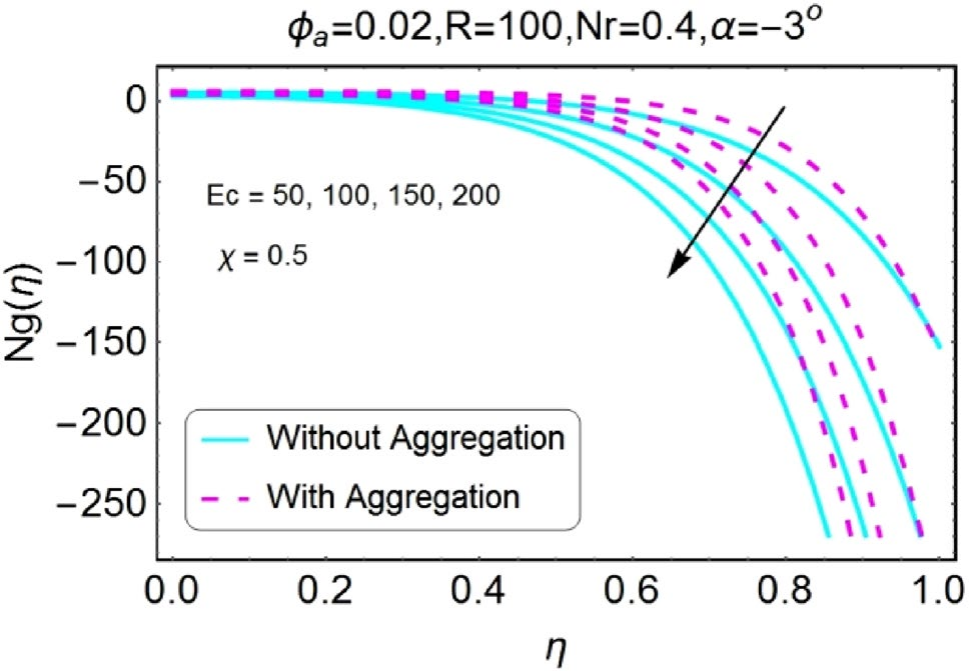
$Ec$ varying $Ng$ for stretching walls (convergent channel).

**Figure 40:**
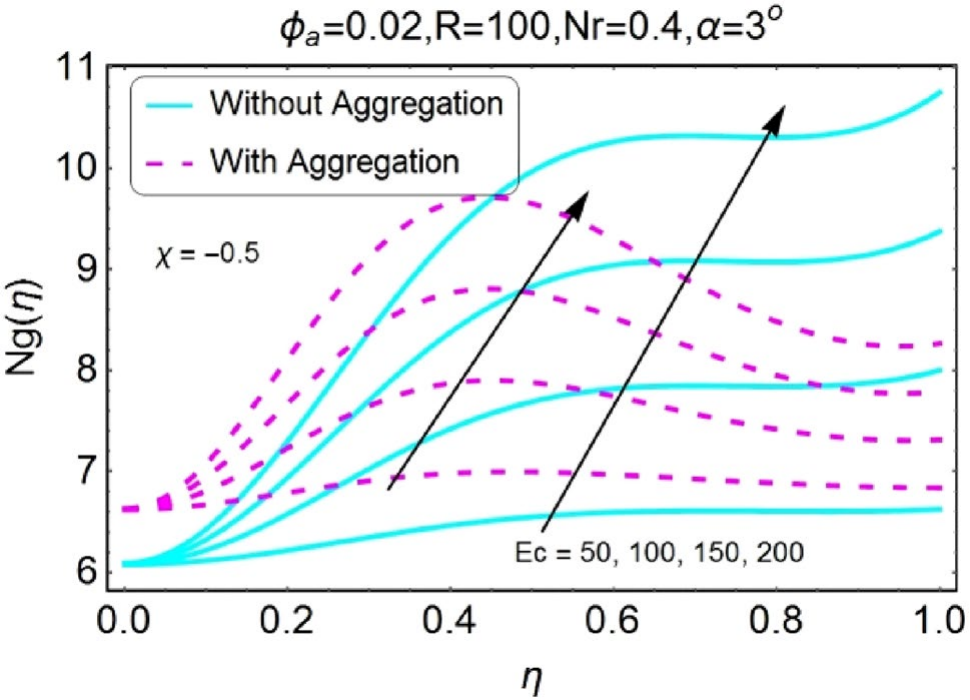
$Ec$ varying $Ng$ for shrinking walls (divergent channel).

**Figure 41:**
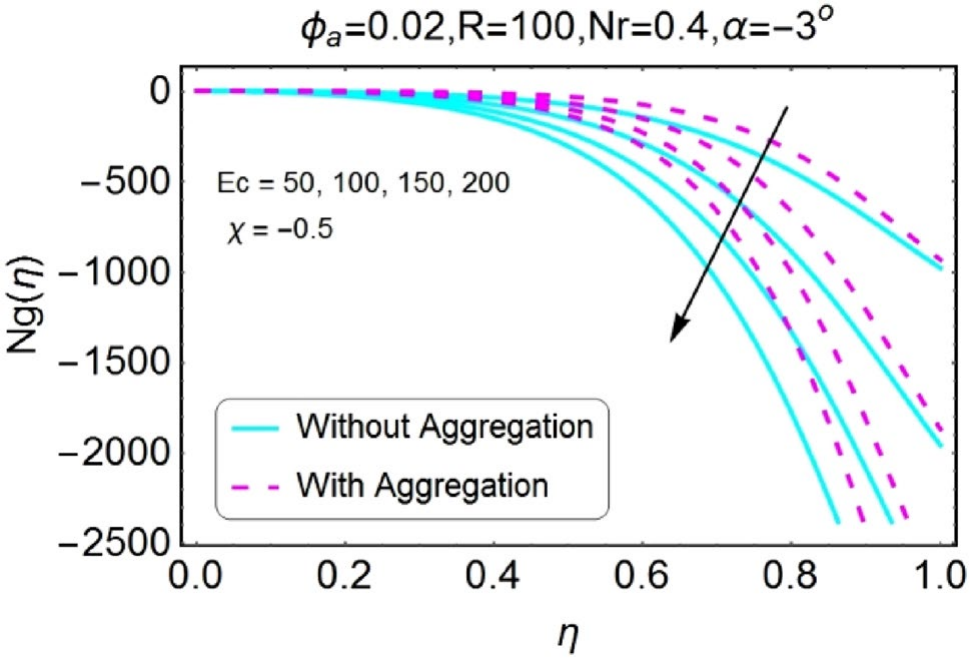
$Ec$ varying $Ng$ for shrinking walls (convergent channel).

**Figure 42:**
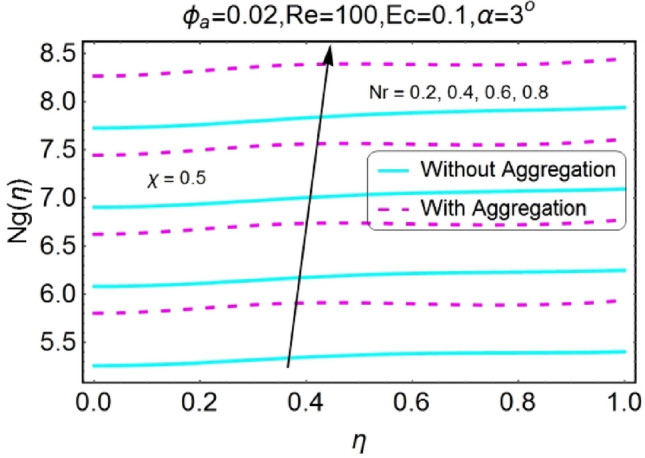
$Nr$ varying $Ng$ for stretching walls (divergent channel).

**Figure 43:**
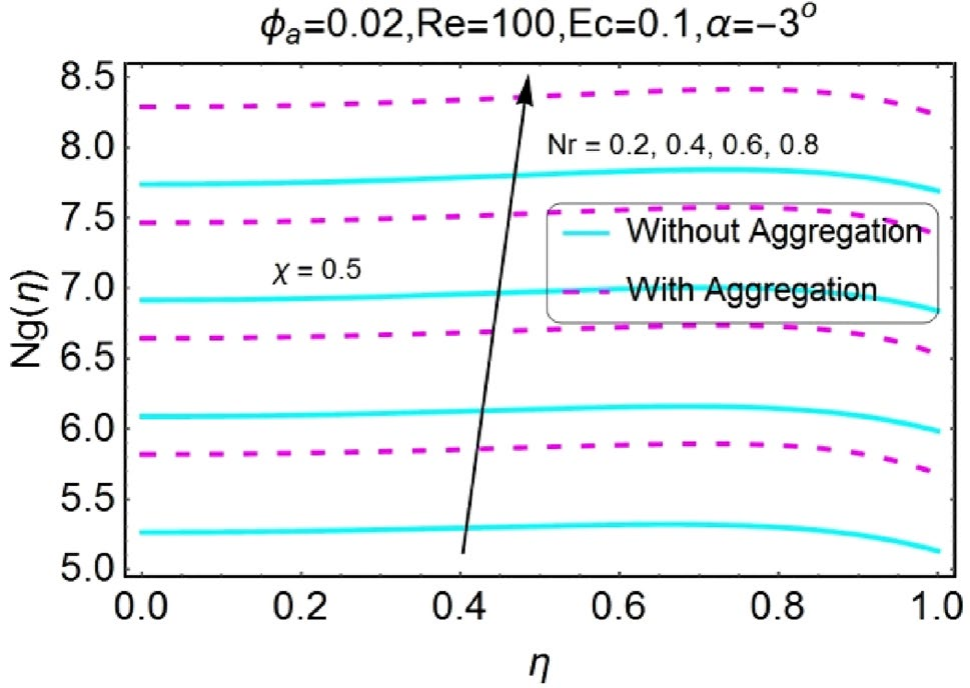
$Nr$ varying $Ng$ for stretching walls (convergent channel).

**Figure 44:**
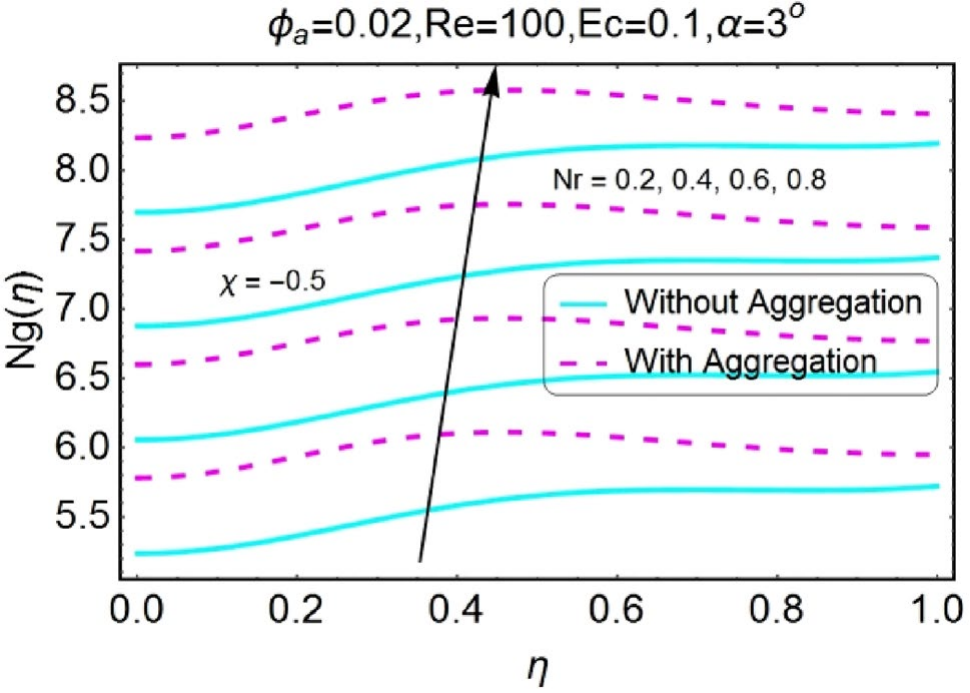
$Nr$ varying $Ng$ for shrinking walls (divergent channel).

**Figure 45:**
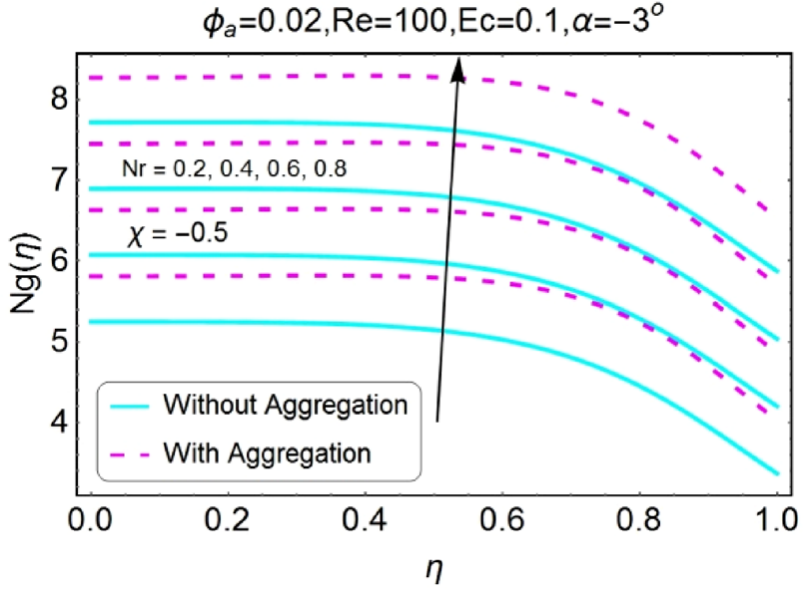
$Nr$ varying $Ng$ for shrinking walls (convergent channel).

**Figure 46:**
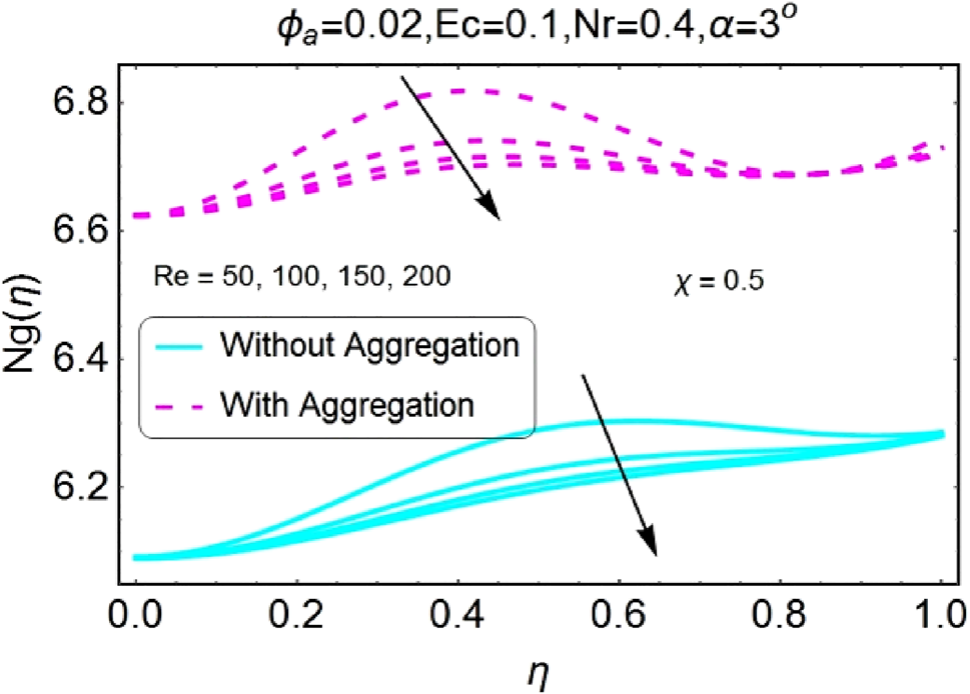
Re varying $Ng$ for stretching walls (divergent channel).

**Figure 47:**
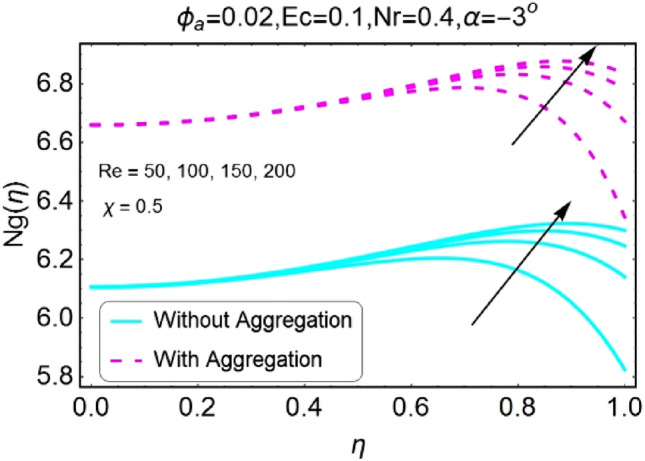
Re varying $Ng$ for stretching walls (convergent channel).

**Figure 48:**
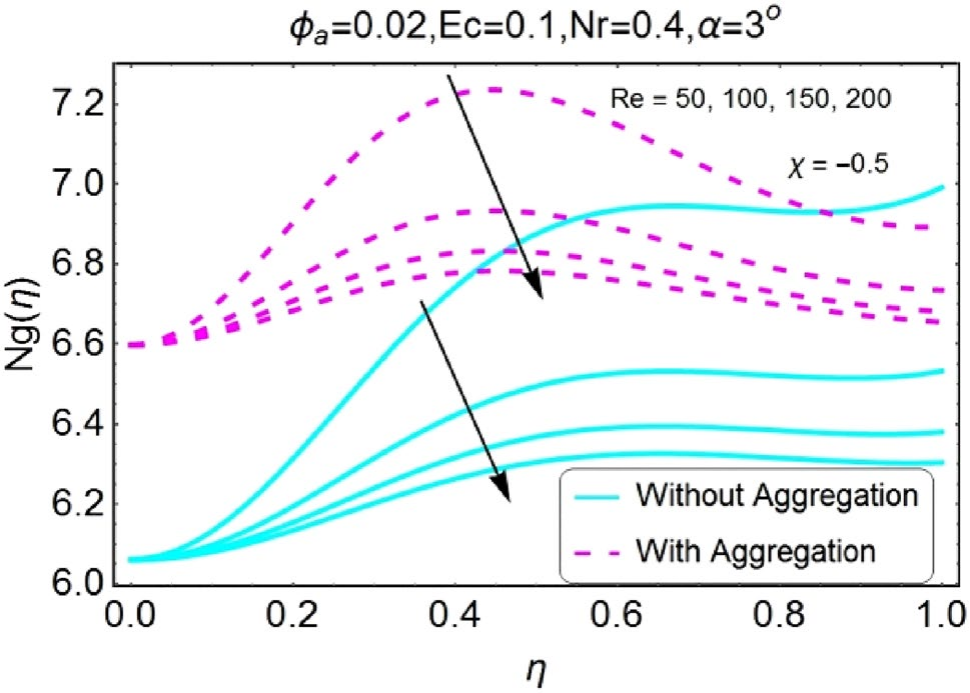
Re varying $Ng$ for shrinking walls (divergent channel).

**Figure 49:**
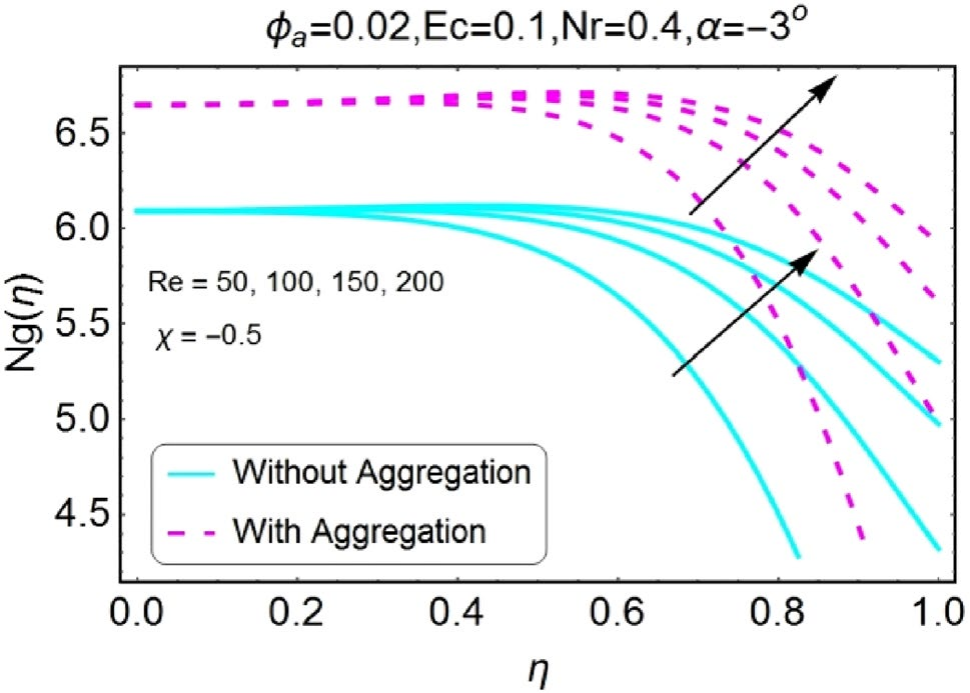
Re varying $Ng$ for shrinking walls (convergent channel).

The impacts of different pertinent parameter like opening angle $(\alpha )$, Eckert number $(Ec)$, Reynolds number $(Re)$ and Radiation parameter $(Nr)$ on entropy generation are portrayed in Figs. 34, 35, 36, 37, 38, 39, 40, 41, 42, 43, 44, 45, 46, 47, 48, 49. The irreversibility of system (entropy generation) reduces with the mounting values of sweep angle $\alpha$ for both converging and diverging channels as shown in Figs. 34, 35, 36, 37. Moreover, the entropy generation profile in the system for stretching walls is slightly above as compared to shrinking walls. The change in irreversibility of system is more prominent closer to the walls of the channels.

The boost in entropy generation profile due to increasing values of Eckert numbers for divergent channel is observed for both stretching/shrinking walls. This alteration in the entropy generation is more prominent near center of channel for stretching/divergent as shown in Fig. 38, whereas for shrinking/divergent channel this change is more effective for aggregated model near center and for non-aggregated model; the change is dominant near the walls as shown in Fig. 40. The entropy generation diminishes by growing the values of $Ec$ in case of converging channel for both stretching and shrinking walls as shown in Figs. 39 and 41. This change in entropy profile is more prominent closer to walls of the channel.

The increase in radiation parameter boosts the entropy generation of the system for all the case involved as shown in the set of Figs. 42, 43, 44, 45. The increasing values of entropy profile for aggregated model are on upper side as compared to non-aggregated model.

The change in entropy generation due to mounting values of Reynolds number is plotted in the set of Figs. 46, 47, 48, 49. Figure 46 shows that there is a drop of entropy generation for growing values of Reynolds number for divergent/stretching channel. This decrement in entropy is prominent near the center of the channel as compared to walls. On the other hand, entropy profile upsurges with the mounting values of Reynolds number $Re$ near the walls for convergent/stretching channel as shown in Fig. 47. From Fig. 48 it is seen that the entropy generation declines for shrinking /divergent channel. It can be observed that for the incrementing values of Reynolds number, the entropy profile increases for shrinking/convergent channel as displayed in Fig. 49. The values of entropy generation for the aggregation model are seems to be on upper side as compared to non-aggregated model for all of the cases shown in Figs. 46, 47, 48, 49.

Table 2 compares the outcomes of the R-K-4 method (together with shooting technique) with the results of the R-K-4 method. Both options are in perfect agreement with one another.

**Table 2:** Comparison between present result and Ref.^8,42^, where ${\phi }_{a}=0, \phi =0.05$.

$\alpha$	Mosta et al.^8^	Rana et al.^42^	Current results
${1}^{o}$	0.9825	0.9825	0.9825
${2}^{o}$	0.9316	0.9316	0.9316
${3}^{o}$	0.8513	0.8513	0.8513
${4}^{o}$	0.7482	0.7482	0.7482

## Conclusion

In the current exploration, the heat transfer and thermal conductivity of steady, incompressible aggregated fluid flow under the effects of magnetic force is considered. The flow is considered between two non-parallel stretching/shrinking walls. The impacts of radiation parameter (Nr), Reynolds number (Re), volume fraction of aggregated nanoparticles (${\phi }_{a}$), Eckert number (Ec) etc. are discussed graphically. The following are the main points of this study:

- The augmenting values of angle of elevation reduces the velocity profile for stretching/shrinking walls of diverging channel, while a reversed performance is detected for the case of converging channel of stretching/shrinking walls.
- The stretching parameter boosts the velocity profile, whereas the shrinking parameter diminishes the velocity field for converging as well as diverging channel.
- The nanoparticles volume friction and Reynolds number declines the velocity profile $f$ for stretching/shrinking walls of divergent channel and boosts the velocity profile for stretching/shrinking walls of convergent channel.
- By mounting the values of sweep angle $\alpha$, the temperature profile rises for all the case i.e., stretching/divergent, shrinking/divergent, stretching/convergent, and shrinking/convergent channels.
- The growing values of $Re$ and $Ec$ upsurges the temperature field for stretching/shrinking divergent channel. On the other hand, for stretching/shrinking convergent channel temperature profile declines by mounting values of these parameters.
- The temperature profile rises for both converging and diverging channels under the effects of stretching parameter, while shrinking parameter diminishes the temperature profile for both converging and diverging channels.
- For the growing values of radiation parameter and nanoparticles volume fraction, the temperature profile reduces for both converging and diverging channels of stretching/shrinking walls.
- The entropy generation declines for all the values of opening angle $\alpha$ (either positive or negative) for the stretching/shrinking walls of channel.
- The increasing values of Eckert number $Ec$ increases the entropy profile for stretching/shrinking divergent channel and decreases for stretching/shrinking convergent channel.
- There is direct variation between radiation parameter and the entropy generation profile i.e., by mounting the values of radiation parameter, irreversibility of the system increases.
- Reynolds number declines the entropy profile for stretching/shrinking diverging channel and upsurges it for stretching/shrinking convergent channel.

## Data Availability

The data and material used and/or analysed during this study are available from the corresponding author on reasonable request.
